# *In vitro* diagnostic methods of Chagas disease in the clinical laboratory: a scoping review

**DOI:** 10.3389/fmicb.2024.1393992

**Published:** 2024-04-30

**Authors:** Luis C. Ascanio, Savannah Carroll, Alberto Paniz-Mondolfi, Juan David Ramírez

**Affiliations:** ^1^Molecular Microbiology Laboratory, Department of Pathology, Molecular and Cell-based Medicine, Icahn School of Medicine at Mount Sinai, New York, NY, United States; ^2^Centro de Investigaciones en Microbiología y Biotecnología-UR (CIMBIUR), Facultad de Ciencias Naturales, Universidad del Rosario, Bogotá, Colombia

**Keywords:** Chagas disease, diagnostic test approval, ELISA, PCR, rapid diagnostic test, review, serology, *Trypanosoma cruzi*

## Abstract

**Background:**

Chagas disease (CD), caused by *Trypanosoma cruzi*, is a global health concern with expanding geographical reach. Despite improved and accessible test methods, diagnosing CD in its various phases remains complex. The existence of clinical scenarios, including immunosuppressed patients, transplant-related CD reactivation, transfusion-associated cases, and orally transmitted acute infections, adds to the diagnostic challenge. No singular gold standard test exists for all phases, and recommendations from PAHO and the CDC advocate for the use of two serological methods for chronic CD diagnosis, while molecular methods or direct parasite detection are suggested for the acute phase. Given the complexity in the diagnostic landscape of CD, the goal of this scoping review is to characterize available diagnostic tests for CD in the clinical laboratory.

**Methods:**

A literature search in PubMed was conducted on studies related to *In vitro* diagnosis (IVD) in humans published in English, Spanish, or Portuguese language as of 28 August 2023, and extended backward with no predefined time frame. Studies underwent title and abstract screening, followed by full-text review. Studies included were classified based on the diagnostic method used. Test methods were grouped as serological, molecular, and other methods. Performance, availability, and regulatory status were also characterized.

**Results:**

Out of 85 studies included in the final review, 115 different tests were identified. These tests comprised 89 serological test types, 21 molecular test types, and 5 other test methods. Predominant serological tests included ELISA (38 studies, 44.70%), Rapid tests (19 studies, 22.35%), and chemiluminescence (10 studies, 11.76%). Among molecular tests, Polymerase Chain Reaction (PCR) assays were notable. Twenty-eight tests were approved globally for IVD or donor testing, all being serological methods. Molecular assays lacked approval for IVD in the United States, with only European and Colombian regulatory acceptance.

**Discussion and conclusion:**

Serological tests, specifically ELISAs, remain the most used and commercially available diagnostic methods. This makes sense considering that most Chagas disease diagnoses occur in the chronic phase and that the WHO gold standard relies on 2 serological tests to establish the diagnosis of chronic Chagas. ELISAs are feasible and relatively low-cost, with good performance with sensitivities ranging between 77.4% and 100%, and with specificities ranging between 84.2% and 100%. Molecular methods allow the detection of specific variants but rely on the parasite’s presence, which limits their utility to parasitemia levels. Depending on the PCR method and the phase of the disease, the sensitivity ranged from 58.88 to 100% while the mean specificity ranged from 68.8% to 100%. Despite their performance, molecular testing remains mostly unavailable for IVD use. Only 3 molecular tests are approved for IVD, which are available only in Europe. Six commercial serological assays approved by the FDA are available for blood and organ donor screening. Currently, there are no guidelines for testing CD oral outbreaks. Although more evidence is needed on how testing methods should be used in special clinical scenarios, a comprehensive approach of clinical assessment and diagnostics tests, including not IVD methods, is required for an accurate CD diagnosis.

## 1 Introduction

Chagas disease (CD) or American Tripanosomiasis caused by the hemoparasite *Trypanosoma cruzi* (*T. cruzi*), is a global health concern as it continues to spread to new areas. It is estimated that it affects between 6 to 8 million people in the Americas, with 70 million people at risk, and an approximate annual incidence of 30,000 to 40,000 cases, 12,000 deaths every year, and 9,000 infected newborns (Nunes et al., 2018; Pan American Health Organization, 2023; World Health Organization, 2023). However, migration from endemic areas, have increased the prevalence of CD in nonendemic countries such as the United States, Canada, Spain, Portugal, Italy, France, Switzerland, Japan, and Australia (Rassi et al., 2010; Manne-Goehler et al., 2016; Nunes et al., 2018; Bern et al., 2020; Velásquez-Ortiz et al., 2022).

*Trypanosoma cruzi* has at least seven discrete typing units (DTUs) which is a genetic classification of the parasite to accommodate the shared genetic features given its genetic diversity (Lima et al., 2015; Velásquez-Ortiz and Ramírez, 2020). The DTUs range from TcI to TcVI, and Tcbat. These confer high genetic and phenotypic diversity which may translate into different clinical presentation and severity, treatment, immune response and ultimately to serological response (Pinazo et al., 2023).

*Trypanosoma cruzi* is mainly transmitted by kissing bugs, hematophagous insects belonging to the subfamily Triatominae. There are 18 genera and 154 species of triatomine bugs (Shi et al., 2020). Triatomines inhabit in tropical and subtropical areas of the Americas, from Southern United States to Argentina, Asia, Africa, and Oceania (Shi et al., 2020; Schijman et al., 2022; Zhao et al., 2023). However, vector transmission only occurs in the Americas. Additionally, CD can result from vertical transmission (4.7%), post-transplant reactivation (75% in hearts, 0%–19% in livers, and kidneys), and transfusion (10%–25% transmission risk) (Hochberg and Montgomery, 2023). Lab infections are rare but can occur accidentally (Nunes et al., 2018; Hochberg and Montgomery, 2023; Pan American Health Organization, 2023). Oral transmission happens through ingesting contaminated food or drinks with triatomine feces containing the parasite (Velásquez-Ortiz and Ramírez, 2020; Hochberg and Montgomery, 2023; World Health Organization, 2023).

Chagas disease has acute and chronic presentations. The acute phase, characterized by high parasitemia, exhibits flu-like symptoms 2–3 weeks post-infection, with varied incubation periods depending on transmission methods (Cantey et al., 2012; Nunes et al., 2018; Velásquez-Ortiz and Ramírez, 2020; Hochberg and Montgomery, 2023). Transfusion and transplant cases may manifest symptoms up to 4 months later (Nunes et al., 2018). Classic signs like Chagoma and Romaña’ sign are less common. Acute fulminant disease can occur in immunosuppressed patients or those infected orally, leading to severe symptoms such as acute myocarditis or death (Nunes et al., 2018; Velásquez-Ortiz and Ramírez, 2020; Candia-Puma et al., 2022; Hochberg and Montgomery, 2023). After 8–12 weeks, the patient transitions to the chronic phase starting with an asymptomatic form with positive infection tests lasting 10–30 years. Ultimately, patients develop cardiac or gastrointestinal symptoms, with approximately 40% developing cardiac disease (Candia-Puma et al., 2022). Gastrointestinal symptoms like megaesophagus or megacolon are less common (Nunes et al., 2018; Velásquez-Ortiz and Ramírez, 2020; Hochberg and Montgomery, 2023). Dysautonomia is documented in the chronic phase, but its role in cardiac pathogenesis remains unknown (Davila et al., 1993; Silva et al., 2022; Moraes et al., 2024; Ribeiro et al., 2024).

Chagas Disease, classified as a neglected tropical disease since 2005 (World Health Organization, 2019, 2023; Centers for Disease Control and Prevention, 2021), incurring with an estimated healthcare associated costs US$627 million (World Health Organization, 2019). The ongoing RAISE project assesses CD and Chagas Cardiomyopathy burden (Andrade et al., 2023; Ribeiro et al., 2024) reported a mean annual hospital cost of 324.44 purchasing power parity (PPP)-USD, a lifetime costs per patients in general care are 209.44 PPP-USD and 14,3451.68 PPPD-USD in patients with heart failure (Andrade et al., 2023). Despite this burden, especially in impoverished areas, its low awareness and complex diagnosing make CD easy to overlook leading to delayed diagnosis and treatment. Among other challenges in diagnosis are its complex clinical presentation complicating confirmation, *T. cruzi’s* genetic diversity reducing test sensitivity, potential false-positive serology from Leishmaniasis cross-reactivity, especially in endemic areas, and consistent migration from endemic to non-endemic areas, elevating prevalence.

Without a universal gold standard, testing for *T. cruzi* includes serological and molecular methods. Direct parasitological methods are reserved for specific scenarios. Molecular tests are preferred in the acute phase, congenital and oral transmission, and in chronic reactivation following immunosuppression (Ramírez et al., 2009; Hernández et al., 2016; Nunes et al., 2018; Hochberg et al., 2021; Forsyth et al., 2022; Pascual-Vázquez et al., 2023; Pinazo et al., 2023). Classical direct parasitological tests can be useful as well. During testing, cross-reactivity with *Lesihmania* spp. (*L.* spp.) and *Trypanosoma rangeli* (*T. rangeli*) is a significant concern, particularly during blood or organ donation, leading to the current “gold standard” of employing two different serological assays in the chronic phase (World Health Organization, 2019; Centers for Disease Control and Prevention, 2021).

While the performance and indications have been documented (Otani et al., 2009; Brasil et al., 2010, 2016; Hernández et al., 2016; Candia-Puma et al., 2022; Suescún-Carrero et al., 2022; Iturra et al., 2023; Pascual-Vázquez et al., 2023), many studies overlook nuances in clinical laboratory diagnosis such as regulatory approval for IVD or donor screening, or market availability. Therefore, understanding the current market offerings of tests is essential for clinicians to choose the right test and for testing laboratories to implement them.

Motivated by these considerations, this study endeavors to conduct a scoping review of all diagnostic tests available in the clinical laboratory for *in vitro* diagnosis (IVD) of Chagas disease. The objective is to characterize the different testing methods used for clinical diagnosis of CD highlighting their indications, regulatory status, performance, and availability. In addition, clinical scenarios such as immunosuppressed patients, CD reactivation post-transplantation, transfusion-associated cases, and oral transmission cases add complexity to the diagnosis (Hernández et al., 2016; Velásquez-Ortiz and Ramírez, 2020; Hochberg et al., 2021).

## 2 Materials and methods

### 2.1 Objectives and outcomes

The main objective is to characterize the existing tests used for the clinical diagnosis of Chagas disease in humans reported in the literature. Secondary objectives included assessment of the status of test performance, availability, indications, and regulatory status as of today. As outcomes, test performance was assessed as sensitivity and specificity. Availability was assessed by cross-referencing the test type identified in the literature with the current manufacturer’s online catalog.

### 2.2 Study query

We performed a scoping review according to the PRISMA-ScR checklist for scoping reviews (Tricco et al., 2018; Supplementary File 1). The literature search was performed in PubMed with the following search terms: “Chagas disease diagnosis,” “Chagas disease diagnosis test,” “Chagas ELISA,” “*T. cruzi* diagnosis,” Chagas commercial diagnosis,” “Chagas diagnosis donor,” “(acute diagnostic test Chagas) AND (acute diagnostic Chagas),” “(congenital Chagas) AND (diagnostic test).” The search covered literature published as of 28 August 2023, and extended backward with no predefined time frame. The query included studies in English, Spanish, and Portuguese language. Studies found outside the initial query that met the inclusion criteria were also included.

Studies were screened by title, abstract, and full-text review. Only studies that met the inclusion criteria were included for analysis. Full text review was performed by two authors (SC and LA) who had assigned a set of studies for screening. Later, reviewers met to verify the studies’ selection and reach a consensus on studies that needed additional screening. The full search strategy is available in Supplementary Table 1.

### 2.3 Study inclusion and exclusion criteria

Inclusion criteria comprised studies conducted in humans where the test type name and manufacturer were disclosed, and the test was utilized for clinical diagnosis. Testing conducted at clinical, or reference laboratories was deemed a surrogate for clinical diagnosis. Additionally, studies without performance assessment were included if they examined a commercial kit or conducted an agreement analysis with an existing assay.

Exclusion criteria encompassed studies not written in English, Spanish, or Portuguese, those involving non-human samples, lacking essential test details such as name or manufacturer, or studies that were unavailable for retrieval. Case reports and case series were also excluded.

### 2.4 Definitions

Studies were classified by test methods as follows: serological methods, molecular methods, and other methods. Other methods groups test types that neither fit as serological or molecular test. Test type refers to the principle of the test.

Serological methods included the following test types: Enzyme-Linked Immunoassay (ELISA), chemiluminescence (CMIA), immunochromatographic rapid diagnostic tests (RDTs), Western-Blot or immunoblot, indirect immunofluorescence (IIF), indirect hemagglutination (IHA), and Radioimmunoprecipitation assay (RIPA).

Molecular test methods included conventional PCR (cPCR), real-time PCR (qPCR), Loop-mediated isothermal amplification (LAMP), and digital droplet PCR (ddPCR). Other tests include direct parasitological methods, or any other method reported in a study that met the inclusion criteria.

Performance was assessed by collecting the sensitivity and specificity values reported for the test in each included study. The mean sensitivity and specificity for each test type was calculated.

Availability refers to commercially available tests. Commercial Tests were classified according to their current existence on the market. A commercial test is defined as any assay marketed for *In vitro* diagnosis (IVD). In-house methods could involve either clinical available in-methods or research methods. Commercial prototypes were also classified as commercial tests. Modified commercial assays were treated as in-house tests.

For commercial tests, regulatory status refers to the approval for a commercial test in the United States, the European Union (EU) or the countries where the test is manufactured and marketed. Commercial test kit inserts, manufacturers’ websites and catalogs, and the FDA website were reviewed to verify their status. Assays labeled as IVD were considered approved in the country of manufacturing. Assays labeled IVD and CE were considered approved for IVD in the EU.

Indications refer to its intended use. For this review, indications were defined as diagnosis of acute Chagas disease, chronic Chagas disease, congenital Chagas, and donor screening. Donor screening refers to tests used to screen for positive CD results in blood donors. Indications were determined depending on the study design and the type of sample used. Studies that analyzed serological methods but did not elaborate on the test’s intended use were assumed to have used the test for Chronic Chagas diagnosis.

### 2.5 Data extraction

The following data was extracted from each included study: study title, authors, test name, test type, test, method, manufacturer, sensitivity, specificity, regulatory status, availability, and indications or intended use. For in-house tests, the name provided will be the study’s first author last name followed by et al.

We extracted sensitivity and specificity values in each study to quantify test performance.

Studies with multiple test types and/or testing methods, duplicate entries were generated for the same study, but each entry will list a single test type and its associated information. The sensitivity and specificity of each test were extracted and used for data analysis.

### 2.6 Critical appraisal

As our study is a scoping review, which typically offers a summary of existing evidence regardless of the quality appraisal, such appraisal is considered optional as long as it aligns with the study aims (Tricco et al., 2018). Since the primary objective of this review was to characterize the available testing types for Chagas disease used for clinical purposes, critical appraisal was not pursued.

### 2.7 Data analysis

Studies were classified based on the diagnostic method and indications. Descriptive statistics were used to report the study findings. Continuous data was reported as mean and standard deviations or median and interquartile range. Categorical data was reported as proportions. Each study was considered to generate a summary of results, counting it once even if it included multiple entries due to various tests assessed. Test types were counted separately. Performance values were manually calculated if not reported in the study but information for calculation was available. In this case, sensitivity and specificity were calculated as True Positive/(True Positive + False Negative) and True Negative/(True Negative + False Positive), respectively.

Mean sensitivity and specificity for each test type were calculated by averaging the individual performance value per test type. For serological methods, mean performance values in each test type group were calculated only for commercial tests available on the market. For molecular and other methods, mean performance was calculated for all test types included with reported performance data, considering the absence of commercial tests in these groups. Data analysis was conducted using Microsoft Excel (Redmond, WA, United States), and figures were generated with Microsoft PowerPoint (Redmond, WA, United States), GraphPad Prism v.10.2.0 (GraphPad Software, LLC), and BioRender (BioRender, Toronto, Canada).

## 3 Results

### 3.1 Summary of included studies

**Figure 1:**
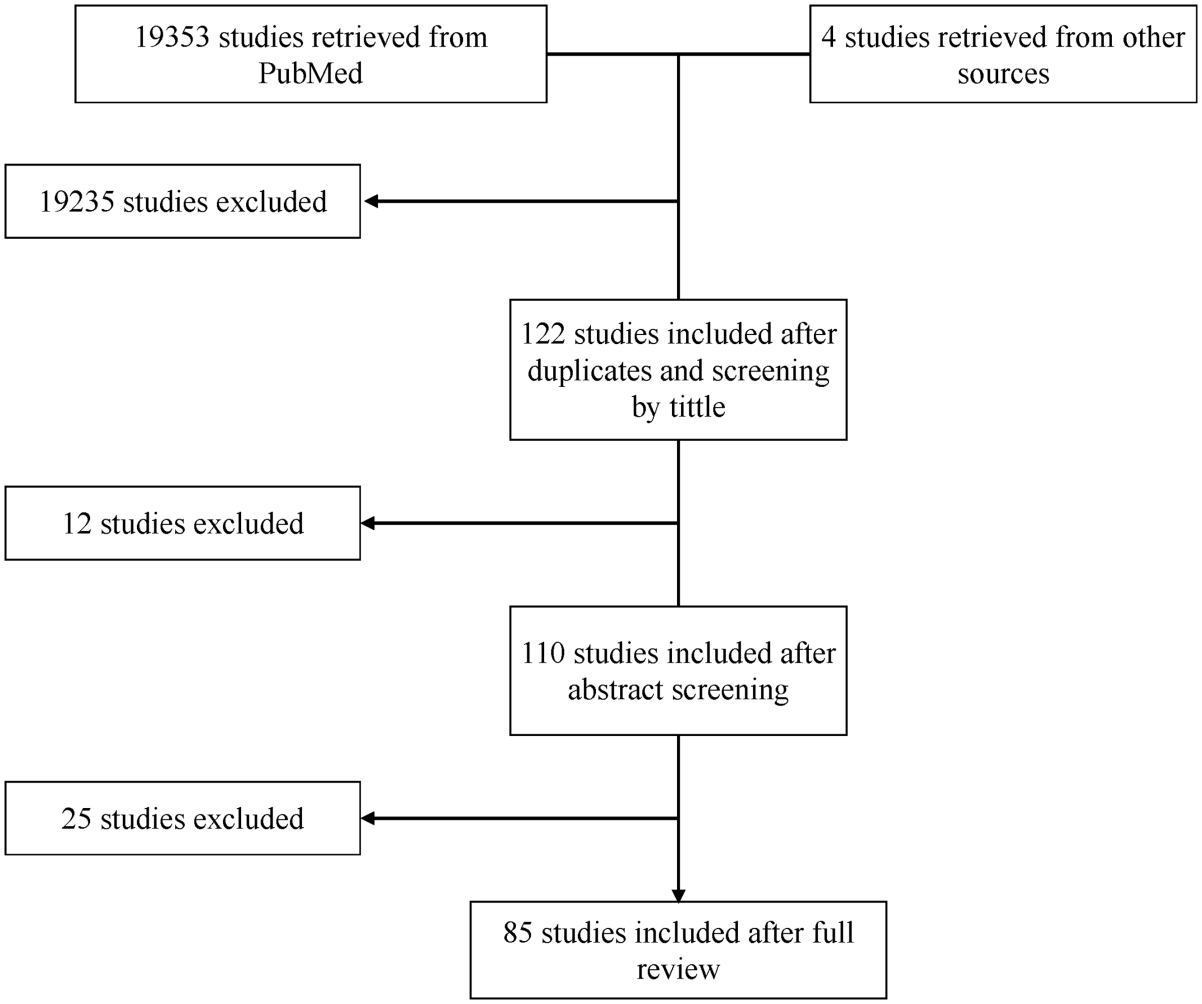
Flowchart of included studies. Initially, 122 studies were considered after title screening and duplicate removal. Subsequently, 12 studies were excluded following abstract screening, resulting in 110 studies for full-text review. After the full-text review, 25 studies were further excluded, leaving 85 studies for comprehensive analysis.

**Figure 2:**
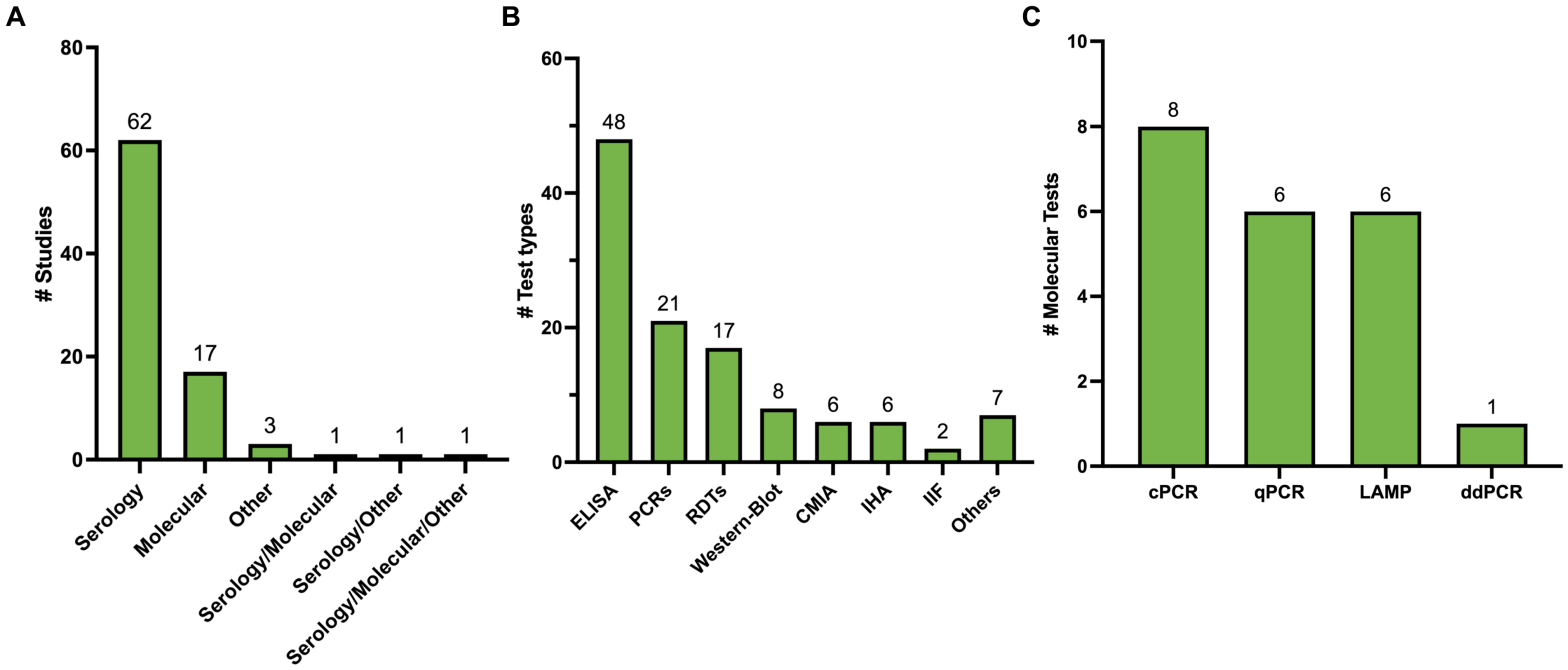
**(A)** Summary of included studies classified by test method. **(B)** Summary of test types identified. Others include 3 direct methods, 1 ELISA/WB combo, 1 RIPA, 1 Flow cytometry, 1 blood culture. **(C)** Summary of type of Molecular Methods Identified.

One-hundred and ten studies underwent full-text review of whom 85 met the inclusion criteria (Figure 1). All included studies are summarized in Supplementary Table 2. Out of 85, 72 studies (84.7%) explored a single test type while 13 studies (15.3%) explored more than a single type. Stratified by testing method, 62 studies (72.94%) covered serological methods (Carvalho et al., 1993; Brashear et al., 1995; Almeida et al., 1997; Hamerschlak et al., 1997; Oelemann et al., 1998; Reiche et al., 1998; Leiby et al., 2000; Ferreira et al., 2001; Ponce et al., 2005; Tobler et al., 2007; Gorlin et al., 2008; Otani et al., 2009; Chappuis et al., 2010; Reithinger et al., 2010; Barfield et al., 2011; Frade et al., 2011; Gamboa-León et al., 2011; Praast et al., 2011; Flores-Chavez et al., 2012; Longhi et al., 2012; Araújo and Berne, 2013; Holguín et al., 2013; Llano et al., 2013; Reis-Cunha et al., 2014; Sánchez-Camargo et al., 2014; Shah et al., 2014; Abras et al., 2016; Santos et al., 2016; Valdez et al., 2016; Angheben et al., 2017; Egüez et al., 2017; Mucci et al., 2017; Cortes-Serra et al., 2018; Flores-Chavez et al., 2018; Mita-Mendoza et al., 2018; Pérez-Ayala et al., 2018; Santos et al., 2018; Kim et al., 2019; Lozano et al., 2019; Mendicino et al., 2019; Whitman et al., 2019; de Oliveira et al., 2020; Brossas et al., 2021; Ferreira-Silva et al., 2021; Hernández et al., 2021; Kelly et al., 2021; Peverengo et al., 2021; Santos et al., 2021; Silgado et al., 2021; Torcoroma-García et al., 2021; Castro-Sesquen et al., 2021a,b,c, Daltro et al., 2022; García-Bermejo et al., 2022; Rivera et al., 2022; Santos et al., 2022; Iturra et al., 2023; Machado et al., 2023; Schaumburg et al., 2023; Moser et al., 2023a,b), 17 studies (20.0%) covered molecular methods (Junqueira et al., 1996; Gomes et al., 1999; Marcon et al., 2002; Virreira et al., 2003; Ramírez et al., 2009; Schijman et al., 2011; Hernández et al., 2016; Besuschio et al., 2017; Abras et al., 2018; Hernández et al., 2018; Ramírez et al., 2018; Wehrendt et al., 2021; Besuschio et al., 2020; Kann et al., 2020; Bisio et al., 2021; Flores-Chavez et al., 2021; Longhi et al., 2023), 3 studies (3.53%) covered other methods (Feilij et al., 1983; Azogue and Darras, 1995; Matos et al., 2011), 1 study (1.18%) covered serology and molecular methods (Duarte et al., 2014), 1 study (1.18%) covered serology and other methods (Pereira et al., 2012), and 1 study (1.18%) covered all 3 methods (Gil-Gallardo et al., 2021) (Figure 2A). Overall, there were 115 tests included for analysis (Figure 2B).

### 3.2 Characteristics of serological methods

Sixty-seven studies yielded 89 serological test types. ELISA was the most studied test type with 38 studies (44.70%) yielding 48 (53.93%) different ELISA assays. Next, RDTs were assessed in 19 studies (22.35%) yielding 17 (19.10%) tests. All these tests were commercially developed. Chemiluminescence was reported in 10 studies (11.76%), yielding six (6.74%) tests. Nine (10.58%) studies explored Western Blot which yielded 8 (9.09%) tests, 4 (3.52%) studies yielded 6 (6.74%) IHA tests, and 3 studies yielded 2 (2.25%) IIF tests, 1 study reported a combined ELISA/Western Blot (1.12%) and 1 study reported RIPA (1.12%; Figure 2B). Table 1 summarizes all commercial serological test methods included. Discontinued and in-house and tests are summarized in Supplementary Tables 3, 4, respectively.

**Table 1:** Characteristics of commercially available serological test included.

#	Commercial test	Manufacturer	Antigen	Use	Regulatory agency approval	Type of Chagas Disease diagnosis	Sensitivity (%)	Specificity (%)
**ELISA**								
1	Chagatest ELISA recombinate v.3.0	Wiener Laboratorios S.A.I.C.	Recombinant	IVD	FDA/EU	Donor/Chronic	96.1	84.2
2	Chagatest ELISA recombinate v.4.0	Wiener Laboratorios S.A.I.C.	Recombinant	IVD	EU/Argentina	Donor/Chronic	98	100
3	Chagatest ELISA lisado	Wiener Laboratorios S.A.I.C.	Lysate	IVD	EU/Argentina	Donor	97.1	100
4	Chagas Detect Fast ELISA	InBios International, Inc.	Recombinant	RUO	N/A	Donor	98.7	98.2
5	Ortho *Trypanosoma cruzi* ELISA Test System	Ortho-Clinical Diagnostics	Lysate	Donor	FDA/EU	Donor/Chronic	96.4	97.4
6	Chagas ELISA IgG + IgM	Vircell S.L.	Recombinant	IVD	EU	Chronic	77.4	100
7	UMELISA CHAGAS	UMELISA	ND	IVD	Cuba	Chronic/Acute	97.7	99.3
8	Chagatek ELISA	Laboratórios Lemos S.R.L.	Lysate	IVD	Argentina	Donor/Chronic	98.1	90.8
9	Biozima Chagas	Laboratórios Lemos S.R.L.	Purified antigens	IVD	Argentina	Chronic	100	94.7
10	Hemagen Chagas’ Kit	Hemagen Diagnostics, Inc.	Purified antigens	IVD	EU	Donor/Chronic	96	95.7
11	Bioelisa Chagas	Werfen, S.A.	ND	IVD	EU	Donor/Chronic	91.7	99.8
12	NovaLisa Chagas	Gold Standard Diagnostics	Recombinant	IVD	EU	Chronic	N/A	N/A
Total							95.2	97.1
**RDT**								
1	Chagas Detect Plus	InBios International, Inc.	Recombinant	IVD	FDA	Donor/Congenital/Chronic	96.51	93.6
2	Chagas STAT PAK Assay	Chembio Diagnostic Systems, Inc.	Recombinant	IVD	EU/Argentina	Donor/Chronic	94.9	98.23
3	WL Check Chagas	Wiener Laboratorios S.A.I.C.	Recombinant	IVD	EU/Argentina	Chronic	92.17	96.47
4	SD BIOLINE Chagas Ab Rapid	Standard Diagnostics, Inc.	Recombinant	IVD	EU	Chronic	92.95	94.42
5	Chagas Ab Combo Rapid Test CE	CTK Biotech, Inc.	Recombinant	IVD	EU	Chronic	91.45	89.15
6	TR Chagas Bio-Manguinhos	Bio-Manguinhos	Recombinant	IVD	EU/Brazil	Chronic	100	78.5
Total							94.66	91.73
**CMIA**								
1	Chagas VirCila (CHR)	Vircell S.L.	Recombinant	IVD	EU	Chronic	98	100
2	Chagas TESA VirCila (TESA)	Vircell S.L.	TESA native	IVD	EU	Chronic	92	100
3	Elecsys Chagas (E-CILA)^*^	Roche Diagnostics	Recombinant FCaBP, FRA, and Cruzipain	IVD	EU/FDA	Donor/ Congenital/ Chronic	98.45	99.95
4	Liaison XL Murex Chagas CILA	DiaSorin S.p.A.	ND	IVD	EU	Donor	76.2	99.5
Total							91.16	99.86
**IHA**								
1	Chagatest IHA	Wiener Laboratorios S.A.I.C.	Lyophilized sheep red blood cells sensitized with *T. cruzi* cytoplasmic antigens	IVD	EU/Argentina	Chronic/Donor	88.9	99.5
2	Chagas HAI Imunoserum (Now HAI Chagas Polychaco)	Laboratórios Lemos S.R.L.	Lyophilized sheep red blood cells sensitized with *T. cruzi* cytoplasmic antigens	IVD	Argentina	Donor	97.6	78.6
3	Imuno-HAI Chagas	Wama Diagnóstica	Red blood cells sensitized with purified antigens of *T. cruzi*	IVD	EU/Brazil	Donor	100	95.8
Total							95.5	91.3
**IFI**								
1	CHAGAS IFI IgG + IgM®	Vircell S.L.	ND	IVD	EU	Congenital	N/A	N/A
**Western Blot**								
1	Chagas Western Blot IgG assay	LDBio Diagnostics	*T. cruzi* larval extract	IVD	EU	Chronic	100	100

^*^Elecsys Chagas received FDA approval for IVD and donor screening on 5 February 2024.

#### 3.2.1 ELISAs

There were 29 (59.18%) commercial ELISA assays identified and 20 (40.82%) in-house ELISA assays identified. Twenty-nine commercial ELISA assays were identified; however, at the time of this review, only 12 (41.38%) commercial ELISA assays remain on the market, in which 10 are approved for IVD, one for donor screening (QuidelOrtho), and one for research use only (InBios). The studies included 6 out of 12 test kits for either chronic Chagas diagnosis or donor screening, 2 only for donor screening, 3 only for screening, and 1 both acute and chronic diagnosis (Table 1).

The sensitivity ranged from 77.4 to 100% with a mean sensitivity of 95.2% and the specificity ranged from 84.2 to 100% and with a mean specificity of 96.4%. All ELISAs, except for UMELISA Chagasfrom UMELISA and Chagas Detect Fast from InBios are approved for use in the EU. In the United States, Chagatest ELISA recombinante v.3.0 from Wiener Lab, the Ortho® *T. cruzi* ELISA Test system from QuidelOrtho, and the Chagas Kit Elisa from Hemagen are the only approved ELISAs by the FDA.

#### 3.2.2 CMIAs

There were 6 chemiluminescence assays identified in our review. These tests are all commercial tests. At the time of the review, the Vircell Microbiologists’s Chagas VirClia®and Chagas TESA VirClia®, the Roche’s Elecsys Chagas, and the DiaSorin’s Liaison XL Murex Chagas CMIAs remain on the market and are approved only in the EU for IVD. Chagas TESA VirClia uses a native TESA antigen, Chagas VirClia uses a recombinant antigen, and Elecsys Chagas uses recombinant antigen representing FCaBP, FRA, and Cruzipain. Their mean sensitivity is 91.16% and the mean specificity is 99.86%. Most tests were used for diagnosis of Chronic CD and for donor screening. The Elecsys Chagas was also used for diagnosis of congenital CD.

#### 3.2.3 RDTs

We found 19 studies that assessed RDTs yielding 17 tests. To the best of our knowledge, only 6 (35.29%) RDTs are currently available while 11 (64.71%) are no longer available. All commercially available RDTs analyzed use recombinant antigens. However, details of these antigens are not disclosed, as they are proprietary.

One test, the PATH-Lemos rapid test (Laboratorio Lemos) was the only prototype rapid test we found, and it is no longer available. Of the six tests, only the Chagas Detect Plus (InBios), is approved by the FDA for IVD. The Chagas Detect Plus (InBios), Stat-Pak (Chembio Diagnostics), and WL Check Chagas (Wiener Lab) are approved for IVD in the EU. The TR Chagas Bio-Manguinhos is approved for IVD only in Brazil.

All six RDTs were used in chronic Chagas and the Chagas Detect Plus was used in congenital samples. The Chagas Detect Plus and the Stat-Pak tests were also used to diagnose donor samples. Overall, the tests’ performance at chronic stage revealed a mean sensitivity is 94.66% and a mean specificity is 91.76% (Table 1).

#### 3.2.4 Western blot assays

There were 8 Western-blot tests identified, 6 were in-house tests, and the remaining 2 were commercial kits. At the time of the review, the Chagas Western Blot IgG assay from LDbio Diagnostics is the only commercial test available (Table 1). This kit is approved only in the EU for IVD, uses Native antigens derived from TcVI genotype, has a sensitivity and specificity of 100% and has been used in the diagnosis of chronic Chagas Disease. The in-house Western blots are summarized in Supplementary Table 3. The other commercial kit, the TESAcruzi, is discontinued (Supplementary Table 3).

#### 3.2.5 IHAs

Six IHA assays were identified, and all were classified as commercial tests. At the time of this review, only half remain commercially available; the Chagatest IHA from Wiener Laboratorios, the HAI Chagas Polychaco from Laboratorios Lemos, and the Immuno-HAI Chagas from WAMA Diagnostica (Table 1). It is worth noting that the HAI Chagas Polychaco was previously called Chagas HAI Imunoserum. All of them are approved for IVD in the EU, Argentina, and Brazil. However, none of these tests are approved by the FDA. Of these tests, the mean sensitivity is 95.5% and the mean specificity 91.3%. Five out of 6 tests were used for donor screening, except for the Chagatest IHA which was also used for Chronic Chagas diagnosis.

#### 3.2.6 IIFs

There were 2 IIF tests identified, the CHAGAS IFI IgG + IgM® from Vircell Microbiologists and the Imunocruzi from Biolab-Merieux. At the time of this review, only the CHAGAS IFI IgG + IgM® remains on the market (Table 1). It is approved in the EU for IVD. Unfortunately, there was only one study that reported its use, and no performance data was available. The study used the test for the diagnosis of congenital Chagas (Table 1).

#### 3.2.7 Other serological tests

There was one study that assessed an in-house RIPA. Performance values were not provided by the authors given that no specimens from parasitological confirmed cases were included. However, its agreement with IIF was 95% (Leiby et al., 2000). RIPA assay was used by the American Red Cross (2024) as a confirmatory method for Chagas disease until 2007. Currently, it replaced its testing with the Ortho *T. cruzi* ELISA and a Rapid test. RIPA is labor intensive, requires parasite culture and radioactive Iodine ^125^I (Leiby et al., 2000); (Supplementary Table 4).

The other study (Valdez et al., 2016) assessed an in-house ELISA/WB combo that used Iron Superoxide dismutase excreted protein as an antigen for the diagnosis of congenital Chagas. While ELISA and Western-Blot tests were performed separately, the authors analyzed them as a single test since there were no statistically significant differences between them. Therefore, the performance values reported involves both test types.

### 3.3 Characteristics of molecular methods

Twenty-one different PCR tests were identified in 19 studies (Figure 2C). Fourteen (66.6%) PCRs were developed in-house, while the remaining five (26.4%) were Loop-mediated Isothermal Amplification (LAMP) prototype tests developed by Eiken Chemical Company. Conventional PCR (cPCR) was the most studied PCR, assessed in eight (38.10%) studies, followed by Real-time PCR (qPCR) in 6 (28.57%) studies, LAMP in 6 (28.57%) studies, and digital PCR (ddPCR) in one study (4.72%). Only one LAMP test was developed in-house. All molecular methods are summarized in Table 2.

**Table 2:** Characteristics and performance of included molecular tests.

Test name	Manufacturer	Sensitivity (%)	Specificity (%)	Target	Type of Chagas Disease diagnosis
**cPCR**					
Gil-Gallardo et al. (2021). In-house cPCR	In-house	N/A	N/A	kDNA	Congenital
Duarte et al. (2014). In-house kDNA PCR	In-house	51.0	100	kDNA	Chronic
Duarte et al. (2014). In-house PCR	In-house	22.0	100	stDNA	Chronic
Ramírez et al. (2009). In-house cPCR	In-house	75.0	100	stDNA	Chronic
Ramírez et al. (2009). In-house PCR	In-house	70.0	100	kDNA	Chronic
Hernández et al. (2016). In-house cPCR	In-house	70.65	98.95	stDNA	Acute/Chronic
Junqueira et al. (1996). In-house cPCR	In-house	N/A	N/A	kDNA	Chronic
Gomes et al. (1999). In-house cPCR	In-house	N/A	N/A	kDNA	Chronic
Marcon et al. (2002). In-house Nested cPCR	In-house	86.0	100	stDNA	Chronic
Schijman et al. (2011). In-house cPCR	In-house	55.9	68.8	kDNA, stDNA, 18S rRNA, 24sα rDNA, SL-DNA, CO II DNA	Donor/ Congenital/ Chronic
Virreira et al. (2003). In-house cPCR	In-house	N/A	N/A	stDNA and kDNA	Congenital
Mean total		58.89	91.94		
**qPCR**					
Kann et al. (2020). Newly Developed One Real-Time Polymerase Chain Reaction (NDO-RT-PCR)	In-house	92.31	100	kDNA, 18S rRNA, stDNA KTZ	Acute/Chronic
Hernández et al. (2016). In-house qPCR	In-house	79.95	98.55	stDNA	Acute/Chronic
RealCyclerCHAG (Abras et al., 2018)	Progenie Molecular—EU approved IVD	N/A	N/A	stDNA	Chronic
Schijman et al. (2011). In-house qPCR	In-house	68.4	77.5	stDNA	Donor/Congenital/Chronic
Hernández et al. (2018). In-house qPCR	In-house	N/A	N/A	stDNA	Acute/Chronic
Mean total		82.84	94.01		
**ddPCR**					
Ramírez et al. (2018). In-house digital droplet (dd)PCR	In-house	100.0	100.0	stDNA	Acute/Chronic
**LAMP**					
Longhi et al. (2023). LAMP: Loopamp LF-160 incubator; PURE: ultrarapid purification system PURE. In-house primers	Eiken Chemical Company	N/A	N/A	stDNA	Chronic
Bisio et al. (2021). In-house LAMP	In-house	69.2	100	18 s rRNA	Congenital
Flores-Chavez et al. (2021). Loopamp *Trypanosoma cruzi* prototype detection kit	Eiken Chemical Company. Prototype	81.5	95.1	stDNA	Congenital/Chronic
Besuschio et al. (2020). Loopamp *Trypanosoma cruzi* prototype kit	Eiken Chemical Company. Protype	93.0	100.0	stDNA	Congenital/Acute/Chronic
Besuschio et al. (2017). Loopamp *Trypanosoma cruzi* prototype kit	Eiken Chemical Company—Prototype	N/A	N/A	stDNA	Acute/Donor/Oral/HIV reactivated/Chronic
Wehrendt et al. (2021). PrintrLab—LAMP *Trypanosoma cruzi* Loopamp prototype kit	Eiken Chemical Company	100.0	100.0	stDNA	Congenital
Mean total		85.92	98.78		

One study highlighted the Progenie Molecular commercial qPCR test, approved for IVD in the EU, but lacked reported performance data (Abras et al., 2018). The study conducted by Hernández et al. (2016), performs an in-house qPCR approved by the Instituto Nacional de Salud (National Institute of Health) of Colombia with a performance of 79.95% Sensitivity and 98.55% Specificity. Reported targets in cPCR were kDNA, stDNA, 18S rRNA, 24sα ribosomal RNA genes (24sα rDNA), spliced-leader DNA genes (SL-DNA), and the subunit II of cytochrome oxidase DNA (CO-II DNA). For qPCR, stDNA was the most common target, while kDNA, 18S rRNA were other targets reported. For ddPCR, stDNA was the only target reported, and LAMP reported stDNA targets as well as 18S rRNA.

Overall, the tests provided indications for diagnosing chronic, acute, and congenital Chagas Disease, or for donor screening. In addition, cPCR, qPCR, and LAMP were used to diagnose congenital Chagas disease.

Only seven (33.3%) assays had their test performance reported, which combined yielded an overall mean sensitivity that ranged from 58.88% to 100%, and a mean specificity from 68.8 to 100%. By type of PCR, ddPCR had the best performance with 100% sensitivity and specificity, followed by qPCR with a mean sensitivity of 82.84% and a mean specificity of 94.01%, and cPCR had the lowest overall performance with 55.88% sensitivity and 91.93% specificity.

### 3.4 Characteristics of other test methods

Among other tests, direct observation by microhematocrit, blood culture, and flow cytometry were identified. All these methods were developed in-house.

Three studies assessed microhematocrit; however, only one study (Azogue and Darras, 1995) reported its performance, with a sensitivity of 49.42% and a specificity of 100% used for the diagnosis of congenital Chagas and the reference test used was placental pathology.

One study (Pereira et al., 2012) used blood culture in donor testing and revealed a sensitivity of 58.07% and a specificity of 100%. Flow cytometry was used for chronic Chagas diagnosis and overall performance was 98.1% sensitivity and 100% specificity (Matos et al., 2011).

## 4 Discussion

**Figure 3:**
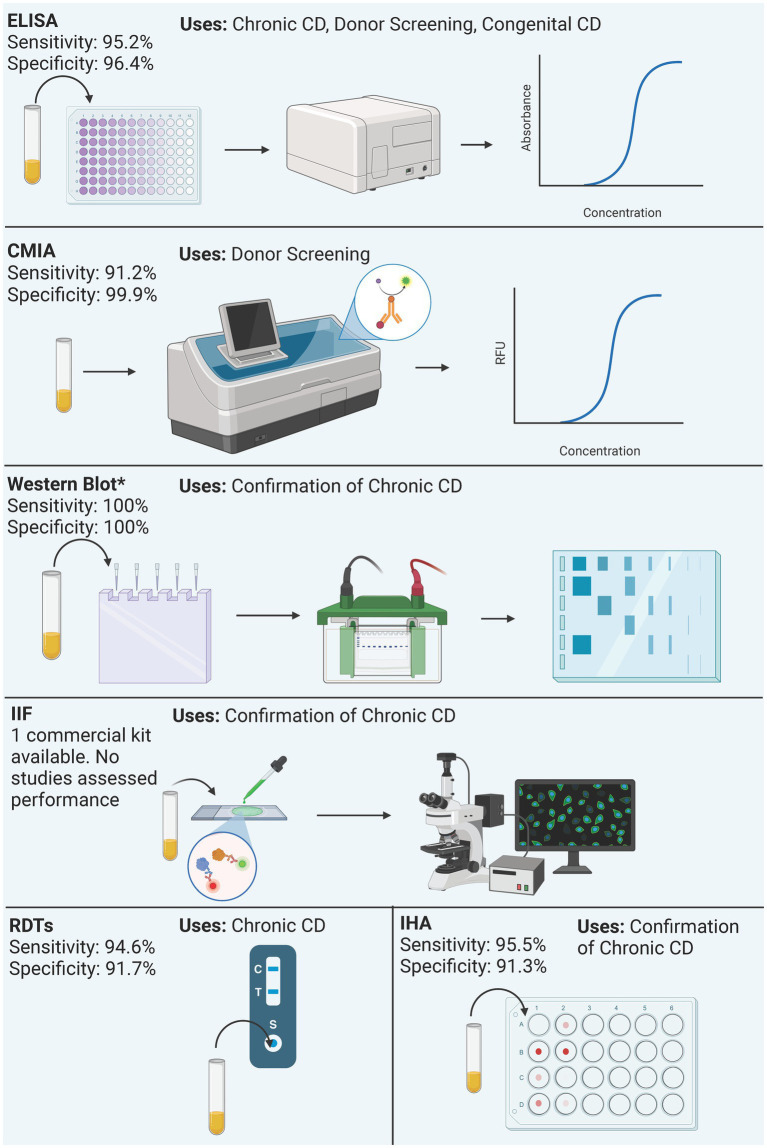
Summary of process and performance of serological methods included in the analysis. Performance is reported as the mean sensitivity and specificity of included studies. Only the performance of commercial kits is reported, regardless of the antigen. Western blot only reports the performance of the Chagas Western Blot IgG assay. ^*^Western Blot performance data derives from a single study.

**Figure 4:**
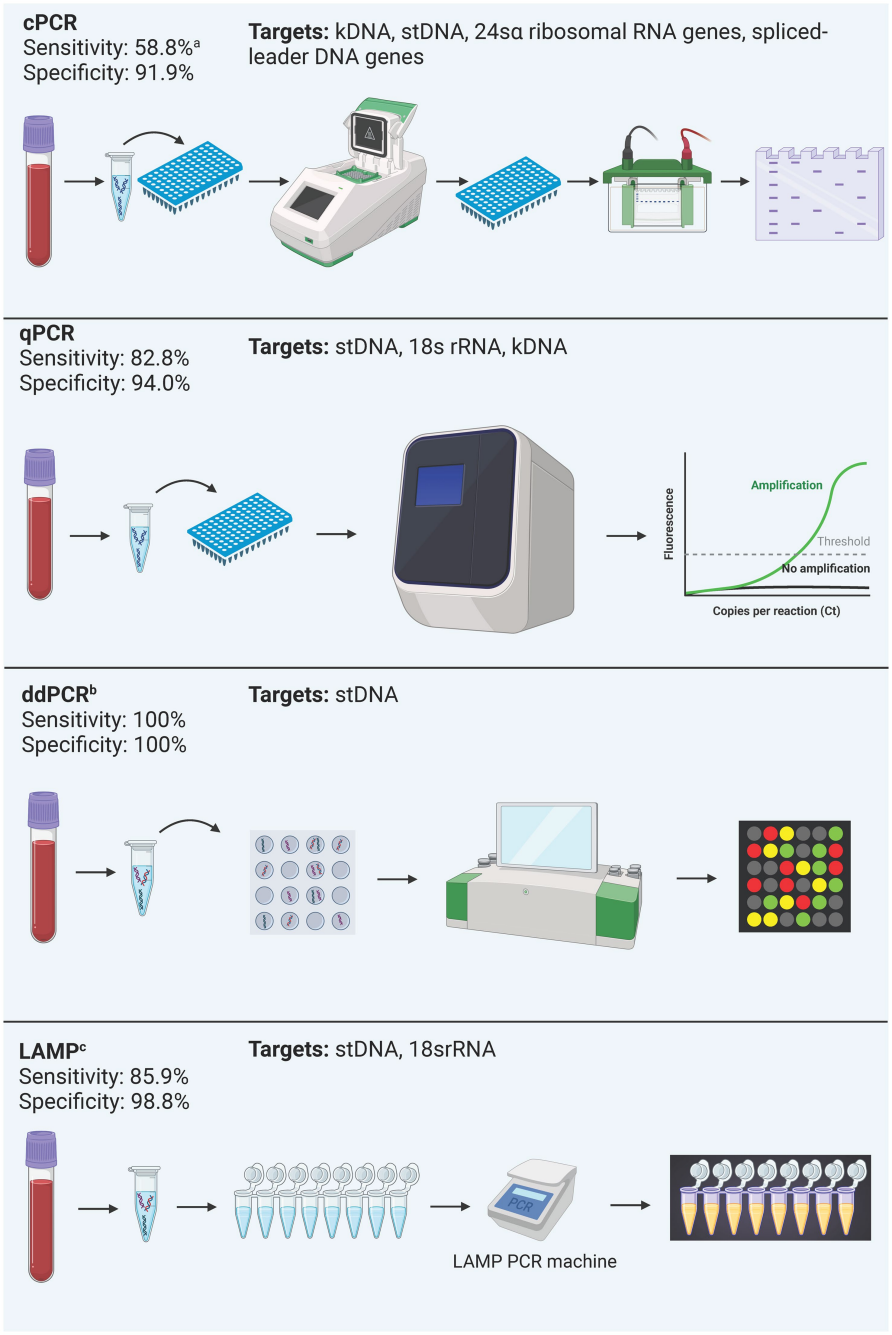
Summary of process and performance of molecular methods included in the analysis. All test types were included regardless of their commercial status due to the little number of commercially available kits. Performance is reported as the mean sensitivity and specificity of included studies. ^a^cPCR: Most studies included used cPCR for chronic Chagas diagnosis, and one included study used cPCR in acute Chagas samples, which explains this sensitivity value (Table 2). ^b^ddPCR: performance data derives from a single study. ^c^LAMP is discontinued.

This review examined 85 studies assessing diagnostic methods for Chagas disease to characterize each test type based on indications, regulatory status, performance, and availability for both clinical and laboratory use. Figures 3, 4 summarizes the process and performance of included serological and molecular methods, respectively. Most studies focused on chronic Chagas samples, reflecting the challenge of detecting the acute stage due to overlapping symptoms with other diseases. Most studies focused on chronic Chagas samples, reflecting the challenge of detecting the acute stage due to overlapping symptoms with other diseases. We identified 28 commercially available tests globally, including ELISAs (*n* = 6), RDTs (*n* = 6), CMIAs (*n* = 6), IHAs (*n* = 3), western blot (*n* = 1), and IIF (*n* = 1). Outside this review, three additional qPCR and one CMIA. However, several tests, particularly ELISAs, RDTs, and IHAs, are discontinued, raising concerns about limited availability for a disease with a significant health burden. Following, we discuss the findings for each test type included in this review.

### 4.1 Serological methods

#### 4.1.1 ELISAs

This review highlights ELISAs as the most utilized method. Our composite performance results are slightly lower from previous meta-analyses (Brasil et al., 2010; Candia-Puma et al., 2022; Suescún-Carrero et al., 2022). These variations in performance may be attributed, in part, to our review’s focus on conducting pooled performance analyses exclusively from commercially available methods. This rational choice resulted in a smaller pool of studies contributing to the overall performance assessment. The rationale behind this decision is to enhance the generalizability of our results since commercial kits are widely accessible across various countries, unlike in-house tests. Moreover, this approach was taken to mitigate bias by excluding test performances from assays that have already been discontinued. This stringent criterion was consistently applied across serological methods included in our review. Despite the differences, the overall performance is comparable, especially among the commercial kits, which are still available to this date. Moreover, DTU genetic diversity may not be responsible for disagreements between serological tests, and rather may be more to individual variation in antibodies’ profiles (Majeau et al., 2024).

ELISAs versatility to multiple assay modifications, allows accommodating various antigens such as *T. cruzi* cell lysate, purified antigens, recombinant antigens, and trypomastigote excreted-secreted antigens (TESA). This feature explains the many ELISAs identified. While the performance of these antigens is comparable (Mita-Mendoza et al., 2018), potential cross-reaction with antigens of *T. rangeli* or *L.* spp. (Caballero et al., 2007) makes them different. Recombinant antigen ELISAs, identified in 50% of commercially available ELISAs in this review, exhibit better specificity against *T. cruzi* and prevent cross-reactivity (Ramírez et al., 2009; Brasil et al., 2010).

Among disadvantages, ELISAs may require manual work, longer turnaround time, the need for skilled personnel and specialized equipment, complex data interpretation, and time-consuming troubleshooting. Moreover, their use is mainly limited to chronic Chagas diagnosis and donor screening.

#### 4.1.2 RDTs

In our review, 17 RDTs were identified, but only six remain on the market, showing a mean sensitivity of 94.66% and a mean specificity of 91.73%, aligning with the findings of Suescún-Carrero et al. (2022). However, our mean specificity was lower primarily attributed to the moderate specificity (78.5%) of the TR Chagas Bio-Manguinhos test. Despite this, the overall specificity remains above 90%. Notably, Chagas Detect Plus is the sole FDA-approved RDT for IVD. All identified RDTs were employed for Chronic Chagas diagnosis, with the Stat-Pak extending its application to donor screening. Similarly, the Chagas Detect Plus was used for donor screening and Congenital Chagas. While these RDTs align with other serological tests for Chronic Chagas diagnosis, their roles in donor screening and congenital cases remain unclear.

Recently, Rivero et al. (2024), assessed the diagnostic performance of four different RDTs, which evaluated two RDTs that were not included in our review due to publication after our query was completed. The first, the ACCU-TELL Chagas Cassette by AccuBiotech Co. Ltd. from China, exhibited a sensitivity of 98% and a specificity of 93%. The second, the Chagas Rapid First Response by Lemos Laboratories from Argentina, demonstrated a sensitivity of 92.5% and a specificity of 96%. Our review already covered the WL Check Chagas and SD Chagas Ab Rapid, which showed higher sensitivities (99% and 100%, respectively) but lower specificities (93% and 76%, respectively) compared to our study’s findings. Notably, the authors could not include the Chagas Detect Plus Rapid Test by InBios in their analysis as it was unavailable for sale at the time of their study was conducted.

Despite their excellent performance, RDTs may be limited to chronic and congenital Chagas diagnosis, especially in isolated areas (World Health Organization, 2019; Hochberg et al., 2021; Forsyth et al., 2022). While PAHO favors ELISA and CMIAs for donor screening due to minimal inaccuracies and substantial cost savings (World Health Organization, 2019), regulatory complexities may also impede RDTs implementation. Moreover, this review highlights a decline in the availability of RDTs for Chagas disease, with only six out of the 17 identified (35.3%) still on the market, emphasizing the importance of financial sustainability for their permanence on the market. Considering these reasons, the role of RDTs in the Chagas disease diagnostic algorithm requires clarification.

#### 4.1.3 IHA

This review found six IHA tests, of which only three are still on the market, exhibiting a mean sensitivity of 95.5% and a mean specificity of 91.3%. Interestingly, all six IHAs were used for donor screening with the Chagatest IHA also employed in chronic Chagas diagnosis. None of the IHAs are FDA-approved, but three are commercially available in Brazil for IVD (Chagatest IHA, HAI Chagas Polychaco, Imuno-HAI Chagas). Chagatest IHA and HAI Chagas Polychaco are also approved for IVD in Argentina. The only test approved for IVD in Europe is the Chagatest IHA. It is worth noting that the HAI Chagas Polychaco was previously called Chagas HAI Immunoserum.

Reader bias and samples with lower reactivity may lead to discrepant results, therefore, the need for careful consideration in its use, especially in large-facility donor screening, must be considered (Otani et al., 2009; Araújo and Berne, 2013). Despite these concerns, PAHO includes IHA as part of the gold standard for chronic Chagas diagnosis (World Health Organization, 2019).

#### 4.1.4 IIF

In this review, we identified three IIF assays, though, only one remains on the market, the CHAGAS IFA IgG + IgM® from Vircell Microbiologist. This test is approved for IVD in Europe. This test was used for diagnosing congenital Chagas in the study by Gil-Gallardo et al. (2021), included in this review. However, performance values for this test were not reported because the study’s focus was on identifying potential positive results for congenital Chagas rather than assessing performance (Gil-Gallardo et al., 2021). It is necessary to include this study because IIFis a commonly used serological technique for diagnosing Chagas disease. IIFs may also be used as part of the diagnostic gold standard for the diagnosis of chronic Chagas (World Health Organization, 2019).

#### 4.1.5 Western blot

We identified eight different Western blot assays, but only one, the Chagas Western Blot IgG assay by LD Diagnostics is commercially available and approved for IVD in Europe. To best of our knowledge, it is the only commercial assay using a specific DTU antigen. This kit in particular is fast and easy to use and contains native extracts from the *T. cruzi* DTU TcVI. In addition, it was successful in confirming discordant results as well as distinguishing between Chagas and *L.* spp. (Brossas et al., 2021). Despite these findings, we did not identify other studies that assessed this kit, so it would be important to determine if the reported sensitivity and specificity reported can be replicated.

The discontinued Western Blot TESAcruzi from BioMeriux used TESA antigens, was FDA-approved (Mita-Mendoza et al., 2018; Daltro et al., 2022), and had 100% sensitivity and 99.16% specificity (Frade et al., 2011). TESA antigens are commonly used in Western Blot due to their high sensitivity and specificity to *T. cruzi* (Ramírez et al., 2009; Frade et al., 2011). Currently, TESA Western blots are confirmatory tests used when two other tests yield conflicting results (Brossas et al., 2021). Despite the high performance, PAHO guidelines (World Health Organization, 2019) do not include them, potentially due to increased diagnostic costs as they are among the most complex serological methods. This complexity may contribute to the limited availability of commercial kits. Therefore, Western Blot is mostly performed in research institutions and reference labs. The CDC employs it for Chagas confirmation.

#### 4.1.6 CMIA

Like ELISA, CMIAs are recommended for the diagnosis of chronic Chagas and donor screening (World Health Organization, 2019). CMIAs use recombinant antigens (Schijman et al., 2022). Typically automated, CMIAs allow higher throughput and quicker turnaround times, making them preferable for large centers and those conducting donor screening. However, they are closed systems with limited room for customization in contrast to ELISA (Cinquanta et al., 2017). Automated instruments, though, come with significant costs, potentially restricting their use in small or underdeveloped labs prevalent in Chagas-endemic regions (World Health Organization, 2019). Furthermore, these instruments come bundled up with other assays as well, which may represent issues for already established labs including old instrument replacements and validations, further incurring logistical and financial burdens.

Of the six identified CMIA assays, four remain on the market. However, it is worth noting that Abbott Laboratories’ FDA-approved assay Abbott PRISM was discontinued and replaced by Alinity S Chagas, another CMIA assay using the proteins FP3, FP6, FP10, and TcF, currently marketed for donor screening in Europe and the United States. There were no studies identified for the Alinity S. Abbott PRISM was marketed outside the United to be used on Abbott Architect (Kelly et al., 2021). For this reason, we analyzed Abbott PRISM and Abbott Architect Chagas as separate assays. On 5 February 2024, Elecsys Chagas received FDA approval for IVD and donor screening. Therefore, as of February 2024, there are five CMIAs on the market.

### 4.2 Molecular methods

Molecular methods enable direct parasite identification by detecting genetic material presence. Common PCR targets for *T. cruzi* include repeat tandem sequence of nuclear DNA or nuclear satellite DNA (stDNA) of DNA sequence E13 and kinetoplast DNA (kDNA; Ramírez et al., 2009; Brasil et al., 2010). Molecular testing also aids in treatment response monitoring, allowing genotypic characterization by identifying DTUs, revealing geographical distribution (Pinazo et al., 2023). However, no clear association exists between DTUs and clinical outcomes or transmission cycles (Velásquez-Ortiz and Ramírez, 2020; Barnabé et al., 2023; Pinazo et al., 2023).

The COVID-19 pandemic changed the landscape for PCR testing, allowing more facilities to perform PCR testing (Pascual-Vázquez et al., 2023; Pinazo et al., 2023). Moreover, the repurposing of PCR platforms used for COVID-19 testing provides an opportunity for centers to testing other for pathogens, including *T. cruzi*. However, the lack of commercial kits poses a challenge for medium and small-size labs due to their limited capabilities to develop an in-house method.

#### 4.2.1 cPCR

Unsurprisingly, cPCR was the most studied PCR method (38.1%), reflecting its historical significance as the first developed and widely performed PCR method (Pinazo et al., 2023). However, its cumulative sensitivity is the lowest among all other PCR methods (58.8%). Most studies included used cPCR for chronic Chagas diagnosis, and one included study used cPCR in acute Chagas samples (Hernández et al., 2016). The lack of guidelines for molecular methods when cPCR became available could explain these findings. It is now known that PCRs are more useful in acute and congenital Chagas even in patients with less than 1 month of age cases due to higher parasitemia (Hernández et al., 2016; Álvarez-Hernández et al., 2018; Pinazo et al., 2023).

The performance varies based on the disease phase when performed, and technical differences, including sample preparation, primers used, and execution (Pinazo et al., 2023). This phenomenon has been reported in a meta-analysis of 21 PCR tests that showed sensitivity values ranging from 50% to 90%, with close to 100% specificity. The differences in performance were attributed to variations in sample storage and preparation. Notably, 20 of these tests were cPCRs and only one was qPCR, which could partly explain the differences observed (Brasil et al., 2010). Moreover, cPCR demonstrated inferior performance compared to qPCR.

#### 4.2.2 qPCR

qPCR offers advantages such as ease of automation, requirement for internal and external controls, and the ability to preserve and hold samples for processing. It has quantitative capabilities and can distinguish between strains (Pinazo et al., 2023). However, its cost limits its availability in endemic areas and demands highly specialized facilities and personnel. Although PAHO does not include qPCR in its testing guidelines, some countries like Chile, Panama, and Argentina consider it an option (Pinazo et al., 2023).

In Chagas disease, qPCR shows over 95% sensitivity (Pinazo et al., 2023), and a meta-analysis reports a median sensitivity of 82.84% and median specificity of 98% (Candia-Puma et al., 2022). However, this review indicates slightly lower mean sensitivity (82.84%) and mean specificity (94.01%). Notably, like cPCR, five of six qPCR assays evaluated performance across varied clinical stages, potentially affecting the results.

Currently, there are three qPCR commercial assays for Chagas: The RealStar Chagas PCR from Werfen, the *T. cruzi* DNA test from Wiener Lab, and the RealCycler CHAG from Progenie Molecular. The *T. cruzi* DNA test reported a sensitivity of 72.73% and a specificity of 99.15% in peripheral venous blood in infants for the diagnosis of congenital Chagas (Benatar et al., 2021). This test is approved for IVD in Argentina and Europe, while the performance of the remaining two tests has not been reported. It is worth nothing that study was discovered subsequent to the initial query and data analysis, resulting in the exclusion of its performance values from the data analysis (Benatar et al., 2021).

#### 4.2.3 ddPCR

Digital droplet PCR (ddPCR) is a novel technique enabling the absolute quantitation of genetic material in a linear manner by partitioning the reaction into droplets in which the proportion of fluorescent droplets in each droplet indicates presence of target and positivity (Ramírez et al., 2018). This method offers advantages such as high target concentration, detection of multiple targets with minimal sample requirement, and excellent performance.

The only study with ddPCR (Ramírez et al., 2018) demonstrated 100% sensitivity and specificity in analyzing both acute and chronic samples, suggesting potential applicability across different clinical stages. Moreover, statistical comparisons of samples between phases revealed no differences. Despite these advantages, limitations include saturation at medium parasite concentrations, lack of clinical validation, and higher costs, hindering widespread clinical use.

#### 4.2.4 LAMP

Loop-mediated isothermal Amplification is a specific molecular method performed under isothermal conditions, requiring a single enzyme. It efficiently amplifies known repetitive sequences shared among different DTUs, allowing visual detection with the naked eye (Besuschio et al., 2017). Developed and marketed in Japan by Eiken Chemical Company, LAMP is currently available for Malaria and Tuberculosis. A prototype for Chagas was once available for research, but it is no longer on the market. LAMP eliminates the need for a thermal cycler, providing results within 1 h (Pinazo et al., 2023). Although more expensive than regular PCR, LAMP offers an overall lower cost per case.

In this review, LAMP was the sole molecular method employed for donor screening, demonstrating acceptable performance in Chagas disease (Besuschio et al., 2017). Overall, LAMP exhibited adequate performance across studies. In comparison to qPCR for chronic Chagas disease, LAMP appears to be more sensitive (Besuschio et al., 2017). Among other uses, LAMP accuracy using FTA cards compared to heparinized blood was 95% (Longhi et al., 2023), however, no performance analyses were performed.

### 4.3 Current status in the US

As of February 2024, the FDA has approved six commercial tests for diagnosing Chagas disease: The Ortho *T. cruzi* ELISA Test System marketed for donor screening, the Wiener Chagatest ELISA recombinante v.3.0, the Hemagen ELISA Chagas Kit, and the InBios RDT Chagas detect plus marketed for IVD, and the Alinity S Chagas and the Elecsys Chagas marketed for both donor screening and IVD. Other countries have more tests available on the market for use including other serological tests and the very few qPCR assays available. The limited number of approved tests in the US raises questions about the disparity compared to other countries. Despite the US’s non-endemic status, the increasing migratory influx from endemic regions poses a risk, emphasizing the importance of effective diagnostic tools, especially in patients who develop chronic Chagas following undetected infection, and in newborns developing congenital Chagas acquired from infected mothers (Hochberg et al., 2021; Forsyth et al., 2022; Hochberg and Montgomery, 2023; Pinazo et al., 2023). The stringent FDA-clearance process, marked by its expense and time-consuming nature, may contribute to the limited number of approved tests. The impact of the COVID-19 pandemic on neglected tropical disease testing further complicates the landscape, diverting resources away from Chagas diagnostics.

Commercial assays are often developed in countries with resources, but low disease prevalence may hinder sustained availability. Argentina and Brazil, where Chagas is endemic, exemplify this contrast with their surveillance programs and numerous test kits, highlighting the challenges in other regions. This is critical as many Chagas tests have been developed but further discontinued, possibly due to lack of profitability for the test developer, which restricts access to CD diagnosis, especially in areas where the disease is endemic (Pinazo et al., 2023).

### 4.4 Testing for treatment response and clinical monitoring

There is currently a significant gap in testing methods for monitoring treatment response in Chagas Disease (Cançado, 1999; Gontijo et al., 1999; Gállego et al., 2020). The traditional standard for confirming clinical cure requires two consecutive negative conventional serology tests (Forsyth et al., 2022). While PCR has been explored as a potential marker for predicting disease progression and response to treatment, continuous negative PCR outcomes do not assure the eradication of parasites (Simon et al., 2020; Sulleiro et al., 2020), as they may persist in tissue-bound sanctuaries (Zhang and Tarleton 1999; Ferreira-Silva et al., 2021). Consequently, alternative approaches such as complement-mediated lysis (CoML) for detecting lytic antibodies have gained attention due to their proven high sensitivity and specificity in identifying anti-*T. cruzi* antibodies during chronic phases (Krettli, 2009). However, using CoML involves handling of live *T. cruzi* parasites, which poses safety concerns in laboratory settings. Thus, the use of purified or recombinant antigens (Guevara et al., 1995; Krettli, 2009) with lytic activity (Krautz et al., 2000) is emerging as a novel approach for diagnosis in an ELISA-based format (Paniz-Mondolfi et al., 2009). Additionally, proteins such as the calcium-binding protein of low molecular weight (Tc24) (Krautz et al., 1995; Paniz-Mondolfi et al., 2009), heat-shock proteins (hsp70) (Krautz et al., 1998), and glycoproteins (GP57/51) (Norris et al., 1991) have shown promising correlation with CoML in assessing response to therapy (Krettli, 2009). Further research into the development of novel biomarkers is necessary to adequately address this important diagnostic gap.

### 4.5 Limitations

Our study focused on the performance of commercially available kits, which could impact the overall performance results. Several direct parasitological methods and xenodiagnosis were not assessed in this study. We attribute this to our focus on studies with performance data available and the exclusion of clinical case reports and case series studies, which usually described these testing methods. Most studies did not use that same reference test to assess its performance. Therefore, the reference performance is often different across studies. This is a potential limitation because the test of interest may seem to perform better if a study’s reference test performs poorly in comparison.

## 5 Conclusion

The diagnostic landscape for Chagas Disease has witnessed advancements in sensitivity and specificity, yet the intricate clinical patterns and the disease’s neglected status continue to present challenges in its diagnosis. Our review provided a comprehensive overview of current diagnostic methods employed in clinical laboratories, their regulatory approval status, and performance characteristics. Notably, serological methods, particularly ELISAs and RDTs, emerged as the predominant tests for clinical diagnosis. While molecular methods showcased utility, their application is primarily limited to acute and high-parasitemia scenarios like Congenital and reactivated Chagas cases. A concerning trend was observed with a decline in the availability of commercial tests, highlighting a growing challenge for this already neglected disease.

## Author contributions

LA: Conceptualization, Data curation, Formal analysis, Investigation, Methodology, Validation, Visualization, Writing – original draft, Writing – review & editing. SC: Data curation, Investigation, Methodology, Validation, Visualization, Writing – original draft, Writing – review & editing. AP-M: Validation, Writing – original draft, Writing – review & editing. JR: Conceptualization, Data curation, Formal analysis, Funding acquisition, Investigation, Methodology, Project administration, Resources, Software, Supervision, Validation, Visualization, Writing – original draft, Writing – review & editing.
