# Supplementary material for: In vitro diagnostic methods of Chagas disease in the clinical laboratory: a scoping review
Source: Front Microbiol. 2024 Apr 30;15:1393992. doi: 10.3389/fmicb.2024.1393992 (PMC11091413; doi:10.3389/fmicb.2024.1393992)
Supplement: Supplementary file 1 [file Table_1.DOCX]

**Supplemental table 1: Electronic query strategy**

| **Search number** | **Query** | **Sort By** | **Filters** | **Search Details** | **Results** | **Time** |
| --- | --- | --- | --- | --- | --- | --- |
| **15** | (congenital Chagas) AND (diagnostic test) |  | from 1000/1/1 - 2023/8/28 | (("congenital"[MeSH Subheading] OR "congenital"[All Fields] OR "congenitally"[All Fields]) AND ("chaga"[All Fields] OR "chaga s"[All Fields] OR "chagas"[All Fields] OR "chagas s"[All Fields]) AND ("diagnostic tests, routine"[MeSH Terms] OR ("diagnostic"[All Fields] AND "tests"[All Fields] AND "routine"[All Fields]) OR "routine diagnostic tests"[All Fields] OR ("diagnostic"[All Fields] AND "test"[All Fields]) OR "diagnostic test"[All Fields])) AND (1000/1/1:2023/8/28[pdat]) | 29 | 23:21:52 |
| **14** | (acute diagnostic test Chagas) AND (acute diagnostic Chagas) |  | from 1000/1/1 - 2023/8/28 | (("acute"[All Fields] OR "acutely"[All Fields] OR "acutes"[All Fields]) AND ("diagnostic tests, routine"[MeSH Terms] OR ("diagnostic"[All Fields] AND "tests"[All Fields] AND "routine"[All Fields]) OR "routine diagnostic tests"[All Fields] OR ("diagnostic"[All Fields] AND "test"[All Fields]) OR "diagnostic test"[All Fields]) AND ("chaga"[All Fields] OR "chaga s"[All Fields] OR "chagas"[All Fields] OR "chagas s"[All Fields]) AND (("acute"[All Fields] OR "acutely"[All Fields] OR "acutes"[All Fields]) AND ("diagnosis"[MeSH Terms] OR "diagnosis"[All Fields] OR "diagnostic"[All Fields] OR "diagnostical"[All Fields] OR "diagnostically"[All Fields] OR "diagnostics"[All Fields]) AND ("chaga"[All Fields] OR "chaga s"[All Fields] OR "chagas"[All Fields] OR "chagas s"[All Fields]))) AND (1000/1/1:2023/8/28[pdat]) | 44 | 23:21:38 |
| **13** | chagas diagnosis donor |  | from 1000/1/1 - 2023/8/28 | (("chaga"[All Fields] OR "chaga s"[All Fields] OR "chagas"[All Fields] OR "chagas s"[All Fields]) AND ("diagnosable"[All Fields] OR "diagnosi"[All Fields] OR "diagnosis"[MeSH Terms] OR "diagnosis"[All Fields] OR "diagnose"[All Fields] OR "diagnosed"[All Fields] OR "diagnoses"[All Fields] OR "diagnosing"[All Fields] OR "diagnosis"[MeSH Subheading]) AND ("donor s"[All Fields] OR "tissue donors"[MeSH Terms] OR ("tissue"[All Fields] AND "donors"[All Fields]) OR "tissue donors"[All Fields] OR "donor"[All Fields] OR "donors"[All Fields])) AND (1000/1/1:2023/8/28[pdat]) | 395 | 23:21:19 |
| **11** | chagas commercial diagnosis |  | from 1000/1/1 - 2018/8/15 | (("chaga"[All Fields] OR "chaga s"[All Fields] OR "chagas"[All Fields] OR "chagas s"[All Fields]) AND ("commercial"[All Fields] OR "commercialism"[All Fields] OR "commercialization"[All Fields] OR "commercialize"[All Fields] OR "commercialized"[All Fields] OR "commercializing"[All Fields] OR "commercially"[All Fields] OR "commercials"[All Fields]) AND ("diagnosable"[All Fields] OR "diagnosi"[All Fields] OR "diagnosis"[MeSH Terms] OR "diagnosis"[All Fields] OR "diagnose"[All Fields] OR "diagnosed"[All Fields] OR "diagnoses"[All Fields] OR "diagnosing"[All Fields] OR "diagnosis"[MeSH Subheading])) AND (1000/1/1:2018/8/15[pdat]) | 120 | 23:20:33 |
| **8** | t cruzi diagnosis |  | from 1000/1/1 - 2023/6/20 | (("chagas disease"[MeSH Terms] OR ("chagas"[All Fields] AND "disease"[All Fields]) OR "chagas disease"[All Fields] OR "t cruzi"[All Fields]) AND ("diagnosable"[All Fields] OR "diagnosi"[All Fields] OR "diagnosis"[MeSH Terms] OR "diagnosis"[All Fields] OR "diagnose"[All Fields] OR "diagnosed"[All Fields] OR "diagnoses"[All Fields] OR "diagnosing"[All Fields] OR "diagnosis"[MeSH Subheading])) AND (1000/1/1:2023/6/20[pdat]) | 8,781 | 23:18:44 |
| **6** | chagas disease diagnosis test |  | from 2018/6/20 - 2023/6/20 | (("chagas disease"[MeSH Terms] OR ("chagas"[All Fields] AND "disease"[All Fields]) OR "chagas disease"[All Fields]) AND ("diagnosable"[All Fields] OR "diagnosi"[All Fields] OR "diagnosis"[MeSH Terms] OR "diagnosis"[All Fields] OR "diagnose"[All Fields] OR "diagnosed"[All Fields] OR "diagnoses"[All Fields] OR "diagnosing"[All Fields] OR "diagnosis"[MeSH Subheading]) AND ("research design"[MeSH Terms] OR ("research"[All Fields] AND "design"[All Fields]) OR "research design"[All Fields] OR "test"[All Fields])) AND (2018/6/20:2023/6/20[pdat]) | 331 | 23:17:50 |
| **4** | chagas ELISA |  | from 1000/1/1 - 2023/6/20 | (("chaga"[All Fields] OR "chaga s"[All Fields] OR "chagas"[All Fields] OR "chagas s"[All Fields]) AND ("elisa s"[All Fields] OR "elisas"[All Fields] OR "enzyme linked immunosorbent assay"[MeSH Terms] OR ("enzyme linked"[All Fields] AND "immunosorbent"[All Fields] AND "assay"[All Fields]) OR "enzyme linked immunosorbent assay"[All Fields] OR "elisa"[All Fields])) AND (1000/1/1:2023/6/20[pdat]) | 1,487 | 23:16:51 |
| **3** | chagas disease diagnosis |  | from 1000/1/1 - 2023/6/20 | (("chagas disease"[MeSH Terms] OR ("chagas"[All Fields] AND "disease"[All Fields]) OR "chagas disease"[All Fields]) AND ("diagnosable"[All Fields] OR "diagnosi"[All Fields] OR "diagnosis"[MeSH Terms] OR "diagnosis"[All Fields] OR "diagnose"[All Fields] OR "diagnosed"[All Fields] OR "diagnoses"[All Fields] OR "diagnosing"[All Fields] OR "diagnosis"[MeSH Subheading])) AND (1000/1/1:2023/6/20[pdat]) | 8,166 | 23:16:43 |
