# Supplementary material for: In vitro diagnostic methods of Chagas disease in the clinical laboratory: a scoping review
Source: Front Microbiol. 2024 Apr 30;15:1393992. doi: 10.3389/fmicb.2024.1393992 (PMC11091413; doi:10.3389/fmicb.2024.1393992)
Supplement: Supplementary file 2 [file Table_2.docx]

Supplemental Table 2. Summary of included studies

| Author | Title | Test Name | Test Type | Manufacturer | Regulatory Agency Approval | Available | Mean Sensitivity | Mean Specificity |
| --- | --- | --- | --- | --- | --- | --- | --- | --- |
| (Abras et al., 2018) | Introducing automation to the molecular diagnosis of Trypanosoma cruzi infection: A comparative study of sample treatments, DNA extraction methods and real-time PCR assays | RealCyclerCHAG | qPCR | Progenie Molecular | EU | Yes | NA | NA |
| (Abras et al., 2016) | Serological Diagnosis of Chronic Chagas Disease: Is It Time for a Change? | ARCHITECT Chagas assay | CMIA | Abbott | EU/ Brazil | No | 100 | 97.6 |
| (Almeida et al., 1997) | A highly sensitive and specific chemiluminescent enzyme-linked immunosorbent assay for diagnosis of active Trypanosoma cruzi infection | Almeida et al. In-house chemiluminescent ELISA | ELISA | NA | NA | NA | 100 | 99.85 |
| (Angheben et al., 2017) | Accuracy of a Rapid Diagnostic Test (Cypress Chagas Quick Test® for the Diagnosis of Chronic Chagas Disease in a Nonendemic Area: A Retrospective Longitudinal Study. | Cypress Chagas Quick Test | RDT | Cypress Diagnostics | EU | No | 82.8 | 98.7 |
| (Araújo and Berne, 2013) | Conventional serological performance in diagnosis of Chagas' disease in southern Brazil | Chagatest | ELISA | Wiener Laboratorios S.A.I.C. | Argentina/EU | Yes | 99.10 | 15.38 |
|  |  | Chagatek ELISA | ELISA | Laboratórios Lemos S.R.L. | Argentina/EU | Yes | 98.3 | 74.35 |
|  |  | EIAgen *T. cruzi* IgG + IgM | ELISA | Adaltis | EU | No | 99.10 | 51.28 |
|  |  | Chagatest IHA | IHA | Wiener Laboratorios S.A.I.C. | EU/Argentina | Yes | 95.9 | 100 |
|  |  | IMUNOCRUZI | IIF | Biolab-Mérieux, S.A. | Brazil/EU | No | 88.5 | 94.8 |
| (Azogue and Darras, 1995) | [Congenital Chagas in Bolivia: comparative study of the effectiveness and cost of diagnostic methods] | Azogue et al. In-house Direct Method | Direct Method | NA | NA | No | 49.43 | 100 |
| (Barfield et al., 2011) | A highly sensitive rapid diagnostic test for Chagas disease that utilizes a recombinant *Trypanosoma cruzi* antigen | PATH-Lemos rapid test (Prototype) | RDT | Laboratórios Lemos S.R.L. | NA | No | 99.5 | 96.8 |
|  |  | Chagas STAT PAK Assay | RDT | Chembio Diagnostic Systems, Inc. | EU/Argentina | Yes | 95.3 | 99.5 |
| Besuschio et al. 2017(Besuschio et al., 2017) | Analytical sensitivity and specificity of a loop-mediated isothermal amplification (LAMP) kit prototype for detection of Trypanosoma cruzi DNA in human blood samples | *Trypanosoma cruzi* Loopamp prototype kit | LAMP | Eiken Chemical Company | NA | No | NA | NA |
| (Besuschio et al., 2020) | *Trypanosoma cruzi* loop-mediated isothermal amplification (Trypanosoma cruzi Loopamp) kit for detection of congenital, acute and Chagas disease reactivation. | *Trypanosoma cruzi* Loopamp prototype kit | LAMP | Eiken Chemical Company | NA | No | 93 | 100 |
| (Bisio et al., 2021) | Diagnostic Accuracy of Two Molecular Tools for Diagnosis of Congenital Chagas Disease. | Bisio et al. In-house LAMP | LAMP | NA | NA | NA | 69.2 | 100 |
|  |  | Bisio et al. In-house qPCR (High Pure PCR Template Preparation Kit) | qPCR | NA | NA | NA | 100 | 100 |
| (Brashear et al., 1995) | Detection of antibodies to Trypanosoma cruzi among blood donors in the southwestern and western United States. I. Evaluation of the sensitivity and specificity of an enzyme immunoassay for detecting antibodies to *T. cruzi* | Brashear et al. In-house Chagas antibody EIA | ELISA | NA | NA | NA | 100 | 99.98 |
| (Brossas et al., 2021) | Evaluation of the Chagas Western Blot IgG Assay for the Diagnosis of Chagas Disease. | Chagas Western Blot IgG assay | Western-Blot | LDBio Diagnostics | EU | Yes | 100 | 100 |
| (Carvalho et al., 1993) | Chagas' disease diagnosis: evaluation of several tests in blood bank screening | Carvalho et al. In-house (CRA + FRA) *cytoplasmic repetitive antigen (CRA) and flagellar repetitive antigen (FRA) | ELISA | NA | NA | NA | 98 | 100 |
|  |  | Abbott Laboratories | ELISA | Abbott | FDA | No | 100 | 98 |
|  |  | Biolab Diagnóstica | ELISA | Biolab Diagnóstica | ND | No | 100 | 96 |
|  |  | Gull Laboratories | ELISA | Gull Laboratories, Inc. | FDA | No | 99 | 98 |
|  |  | ORTHO *T. cruzi* ELISA Test System | ELISA | Ortho-Clinical Diagnostics | FDA/EU | Yes | 99 | 95 |
| (Castro-Sesquen et al., 2021c) | The Immunoglobulin M-Shed Acute Phase Antigen (SAPA)-test for the Early Diagnosis of Congenital Chagas Disease in the Time of the Elimination Goal of Mother-to-Child Transmission. | Castro-Sesquen et al.2021c Immunoglobulin M (IgM)-Shed Acute Phase Antigen (SAPA) test | ELISA | NA | NA | NA | 76.7 | 98.9 |
| (Castro-Sesquen et al., 2021b) | Evaluation of 2 Lateral Flow Rapid Tests in the Diagnosis of Chagas Disease in the Washington Metropolitan Area. | Chagas Detect Plus | RDT | InBios International, Inc. | FDA | Yes | 91.9 | 80.3 |
|  |  | Chagas STAT PAK Assay | RDT | Chembio Diagnostic Systems, Inc. | EU/Argentina | Yes | 89.7 | 97.1 |
| (Castro-Sesquen et al., 2021a) | Use of a Latent Class Analysis in the Diagnosis of Chronic Chagas Disease in the Washington Metropolitan Area. | Chagatest ELISA recombinate v.3.0 | ELISA | Wiener Laboratorios S.A.I.C | FDA | Yes | 94.9 | 99.6 |
|  |  | Hemagen Chagas’ Kit | ELISA | Hemagen Diagnostics, Inc. | EU | Yes | 100 | 90.4 |
|  |  | Chagas Detect Plus | RDT | InBios International, Inc. | FDA | Yes | 93.6 | 94.7 |
|  |  | Castro-Sesquen et al.2021a In-house IgG-TESA-blot | Western-Blot | NA | NA | NA | 100 | 98.7 |
| (Chappuis et al., 2010) | Validation of a rapid immunochromatographic assay for diagnosis of Trypanosoma cruzi infection among Latin-American Migrants in Geneva, Switzerland | Chagas STAT PAK Assay | RDT | Chembio Diagnostic Systems, Inc. | EU/Argentina | Yes | 95.6 | 99.85 |
| (Cortes-Serra et al., 2018) | Diagnosis of Trypanosoma cruzi Infection Status using Saliva of Infected Subjects | Bioelisa Chagas | ELISA | Werfen, S.A. | EU | Yes | 70 | 100 |
|  |  | DRG *Trypanosoma cruzi* IgG | ELISA | DRG International, Inc. | ND | No | ND | ND |
| (Daltro et al., 2022) | Western blot using Trypanosoma cruzi chimeric recombinant proteins for the serodiagnosis of chronic Chagas disease: A proof-of-concept study. | Daltro et al. 2022 In-house Western blot | Western-Blot | NA | NA | NA | 98.75 | 100 |
| (de Oliveira et al., 2020) | ELISA Saliva for Trypanosoma cruzi Antibody Detection: An Alternative for Serological Surveys in Endemic Regions. | Chagatest ELISA recombinate v.4.0 | ELISA | Wiener Laboratorios S.A.I.C. | EU/ Argentina | Yes | 97 | 100 |
| (Duarte et al., 2014) | Comparison of seven diagnostic tests to detect Trypanosoma cruzi infection in patients in chronic phase of Chagas disease. | Duarte et al. In-house kDNA PCR | cPCR | NA | NA | NA | 51 | 100 |
|  |  | Duarte et al. In-house nDNA PCR | cPCR | NA | NA | NA | 22 | 100 |
|  |  | Duarte et al. In-house ELISA | ELISA | NA | NA | NA | 93 | 100 |
|  |  | Bioelisa Chagas | ELISA | Werfen, S.A. | EU | Yes | 98 | 100 |
|  |  | Chagatest ELISA recombinate v.3.0 | ELISA | Wiener Laboratorios S.A.I.C. | FDA | Yes | 90 | 100 |
|  |  | Chagatest IHA | IHA | Wiener Laboratorios S.A.I.C. | EU/Argentina | Yes | 73 | 100 |
|  |  | SD BIOLINE Chagas Ab Rapid | RDT | Standard Diagnostics, Inc.(Now Abbott) | EU | Yes | 88 | 100 |
| (Egüez et al., 2017) | Rapid diagnostic tests duo as alternative to conventional serological assays for conclusive Chagas disease diagnosis. | Chagas STAT PAK Assay | RDT | Chembio Diagnostic Systems, Inc. | EU/Argentina | Yes | 100 | 99.3 |
|  |  | Chagas Detect Plus | RDT | InBios International, Inc. | FDA | Yes | 100 | 99.3 |
| (Feilij et al., 1983) | Direct micromethod for diagnosis of acute and congenital Chagas' disease | Feilij et al. In-house Direct Method | Direct Method | NA | NA | NA | NA | NA |
| (Ferreira et al., 2001) | Enzyme-linked immunosorbent assay for serological diagnosis of Chagas' disease employing a Trypanosoma cruzi recombinant antigen that consists of four different peptides. | Ferreira et al. TcF-ELISA | ELISA | NA | NA | NA | 100 | 98.94 |
| (Ferreira-Silva et al., 2021) | Chagas disease: Performance analysis of immunodiagnostic tests anti-Trypanosoma cruzi in blood donors with inconclusive screening results. | Ferreira-Silva et al. In-house ELISA-IgG1 and ELISA-IgG3 using Wiener 3.0 | ELISA | NA | NA | NA | 97.94 | 80.09 |
|  |  | Ferreira-Silva et al. In-house ELISA-rCRP | ELISA | NA | NA |  | NA | NA |
|  |  | Chagatek ELISA | ELISA | Laboratório Lemos S.R.L. | Argentina | Yes | 96.64 | 98.77 |
|  |  | Chagatest ELISA recombinate v.3.0 | ELISA | Wiener Laboratorios S.A.I.C. | FDA | Yes | 97.91 | 78.08 |
|  |  | Ferreira-Silva et al. In-house TESA-blot | Western-Blot | NA | NA | NA | 97.72 | 98.07 |
| (Flores-Chavez et al., 2018) | Evaluation of the Elecsys Chagas Assay for Detection of Trypanosoma cruzi-Specific Antibodies in a Multicenter Study in Europe and Latin America | Elecsys Chagas (E-CILA: Electrochemiluminiscence)** | CMIA | Roche Diagnostics | EU | Yes | 100 | 99.89 |
| (Flores-Chavez et al., 2021) | Evaluation of the Performance of the Loopamp Trypanosoma cruzi Detection Kit for the Diagnosis of Chagas Disease in an Area Where It Is Not Endemic, Spain. | Trypanosoma cruzi Loopamp prototype kit | LAMP | Eiken Chemical Company | NA | No | 81.5 | 95.1 |
| (Flores-Chavez et al., 2012) | Sensitivity and specificity of an operon immunochromatographic test in serum and whole-blood samples for the diagnosis of Trypanosoma cruzi infection in Spain, an area of nonendemicity. | Operon immunochromatographic test (ICT-Operon; Simple Stick Chagas) | RDT | Operon, S.A. | ND | No | 94.625 | 94.525 |
|  |  | Operon immunochromatographic test (ICT-Operon Simple Chagas WB [whole blood]) | RDT | Operon, S.A. | ND | No | 94.625 | 94.525 |
| (Frade et al., 2011) | Western blotting method (TESAcruzi) as a supplemental test for confirming the presence of anti-Trypanosoma cruzi antibodies in finger prick blood samples from children aged 0-5 years in Brazil | TESAcruzi | Western-Blot | Biolab-Mérieux, S.A. | Brazil | No | 100 | 99.16 |
| (Gamboa-León et al., 2011) | Do commercial serologic tests for Trypanosoma cruzi infection detect Mexican strains in women and newborns? | Gamboa-Leon et al. In-house ELISA using strain of *T. cruzi* that is prevalent in Mexico (I) | ELISA | NA | NA | NA | NA | NA |
| (García-Bermejo et al., 2022) | Evaluation of the Chagas VirClia(R) and Chagas TESA VirClia(R) for the Diagnosis of Trypanosoma cruzi Infection. | Chagas VirCila (CHR) | CMIA | Vircell S.L. | EU | Yes | 98 | 100 |
|  |  | Chagas TESA VirCila (TESA) | CMIA | Vircell S.L. | EU | Yes | 92 | 100 |
| (Gil-Gallardo et al., 2021) | Chemiluminescent Microparticle Immunoassay for the Diagnosis of Congenital Chagas Disease: A Prospective Study in Spain. | CMIA Architect Chagas (chemiluminescent microparticle immunoassays (CMIA) | CMIA | Abbott | EU | No | NA | NA |
|  |  | Gil-Gallardo et al. In-house conventional PCR | cPCR | NA | NA | NA | NA | NA |
|  |  | Gil-Gallardo et al. In-house Direct Method | Direct Method | NA | NA |  | NA | NA |
|  |  | CHAGAS IFI IgG+IgM® | IIF | Vircell S.L. | EU | Yes | NA | NA |
| (Gomes et al., 1999) | Chagas' disease diagnosis: comparative analysis of parasitologic, molecular, and serologic methods | Gomes et al. In-house cPCR | cPCR | NA | NA | NA | NA | NA |
| (Gorlin et al., 2008) | Evaluation of a new Trypanosoma cruzi antibody assay for blood donor screening. | Ortho *T. cruzi* ELISA Test System | ELISA | Ortho-Clinical Diagnostics | FDA/ EU | Yes | 99.45 | 99.699 |
| (Hamerschlak et al., 1997) | Chagas' disease: an algorithm for donor screening and positive donor counseling | Abbott | ELISA | Abbott | No | FDA | 100 | 95 |
|  |  | Biolab-Mérieux | ELISA | Biolab-Mérieux, S.A. | No | Brazil | 100 | 87 |
|  |  | Gull | ELISA | Gull Laboratories, Inc. | No | FDA | 100 | 97 |
| (Hernández et al., 2021) | Evaluation of UMELISA CHAGAS™ with the incorporation of new monomeric and chimeric peptides representative of different regions of Trypanosoma cruzi | UMELISA CHAGAS | ELISA | UMELISA | Cuba | Yes | 97.73 | 99.33 |
| (Hernández et al., 2018) | Comparison of parasite loads in serum and blood samples from patients in acute and chronic phases of Chagas disease | Hernández et al. In-house cPCR | cPCR | NA | NA | NA | NA | NA |
| (Hernández et al., 2016) | Molecular Diagnosis of Chagas Disease in Colombia: Parasitic Loads and Discrete Typing Units in Patients from Acute and Chronic Phases. | Hernández et al.  In-house qPCR | qPCR | NA | NA | NA | 79.95 | 98.55 |
|  |  | Hernández et al.  In-house PCR | cPCR | NA | NA | NA | 70.65 | 98.95 |
| (Holguín et al., 2013) | Dried blood as an alternative to plasma or serum for Trypanosoma cruzi IgG detection in screening programs. | ARCHITECT Chagas assay | CMIA | Abbott | EU/ Brazil | No | 97 | 77.85 |
| (Iturra et al., 2023) | A multicenter comparative study of the performance of four rapid immunochromatographic tests for the detection of anti-Trypanosoma cruzi antibodies in Brazil. | Chagas Ab Combo Rapid Test CE | RDT | CTK Biotech, Inc. | EU | Yes | 92.8 | 87.3 |
|  |  | SD BIOLINE Chagas Ab Rapid | RDT | Standard Diagnostics, Inc.(Now Abbott Laboratories) | EU | Yes | 95.5 | 89.9 |
|  |  | WL Check Chagas | RDT | Wiener Laboratorios S.A.I.C. | EU/Argentina | Yes | 97.3 | 92.4 |
|  |  | TR Chagas Bio-Manguinhos | RDT | Bio-Manguinhos | EU/Brazil | Yes | 100 | 78.5 |
| (Junqueira et al., 1996) | Comparison of the polymerase chain reaction with two classical parasitological methods for the diagnosis of Chagas disease in an endemic region of north-eastern Brazil | Junqueira et al. In-house cPCR | cPCR | NA | NA | NA | NA | NA |
| (Kann et al., 2020) | Chagas Disease: Detection of Trypanosoma cruzi by a New, High-Specific Real Time PCR. | Kann et al. In-house Newly Developed One Real-Time Polymerase Chain Reaction (NDO-RT-PCR) | qPCR | NA | NA | NA | 92.31 | 100 |
| (Kelly et al., 2021) | Comparative Performance of Latest-Generation and FDA-Cleared Serology Tests for the Diagnosis of Chagas Disease. | Abbott PRISM Chagas | CMIA | Abbott | FDA | No | 95.5 | 100 |
|  |  | Chagatest ELISA lisado | ELISA | Wiener Laboratorios S.A.I.C. | EU/Argentina | Yes | 97.1 | 100 |
|  |  | Chagatest ELISA recombinate v.4.0 | ELISA | Wiener Laboratorios S.A.I.C. | EU/Argentina | Yes | 98.9 | 100 |
| (Kim et al., 2019) | Detection of Human Anti-Trypanosoma cruzi Antibody with Recombinant Fragmented Ribosomal P Protein. | Kim et al. Fragmented Ribosomal P protein | Western-Blot | NA | NA | NA | NA | NA |
| (Leiby et al., 2000) | Serologic testing for Trypanosoma cruzi: comparison of radioimmunoprecipitation assay (RIPA) with commercially available indirect immunofluorescence assay, indirect hemagglutination assay, and enzyme-linked immunosorbent assay kits | Leiby et al. In-House radioimmunoprecipitation assay (RIPA) | RIPA | NA | NA | NA | NA | NA |
| (Llano et al., 2014) | [Preliminary evaluation of the commercial kit Chagas ( Trypanosoma cruzi ) IgG-ELISA ¬Æ in Colombian individuals]. | Novalisa Chagas | ELISA | Gold Standard Diagnostics | EU | Yes | NA | NA |
| (Longhi et al., 2012) | Evaluation of in-house ELISA using Trypanosoma cruzi lysate and recombinant antigens for diagnosis of Chagas disease and discrimination of its clinical forms. | Longhi et al. in-house ELISA | ELISA | NA | NA | NA | 63.56 | 87.41 |
| (Longhi et al., 2023) | Combination of ultra-rapid DNA purification (PURE) and loop-mediated isothermal amplification (LAMP) for rapid detection of Trypanosoma cruzi DNA in dried blood spots. | LAMP: Loopamp LF-160 incubator; PURE: ultrarapid purification system PURE. In-house primers | LAMP | Eiken Chemical Company | NA | No | NA | NA |
| (Lozano et al., 2019) | Use of rapid diagnostic tests (RDTs) for conclusive diagnosis of chronic Chagas disease - field implementation in the Bolivian Chaco region | Chagas STAT PAK Assay | RDT | Chembio Diagnostic Systems, Inc. | EU/Argentina | Yes | 97.7 | 97.4 |
|  |  | Chagas Detect Plus | RDT | InBios International, Inc. | FDA | Yes | 98.4 | 87.1 |
| (Machado et al., 2023) | Proof of Concept of a Novel Multiepitope Recombinant Protein for the Serodiagnosis of Patients with Chagas Disease. | Machado et al. 2023 In-house ELISA with rTC (recomb protein antigen) | ELISA | NA | NA | NA | 98.28 | 96.67 |
| (Marcon et al., 2002) | Use of a nested polymerase chain reaction (N-PCR) to detect Trypanosoma cruzi in blood samples from chronic chagasic patients and patients with doubtful serologies | Marcon et al. 2002 In-house Nested PCR | cPCR | NA | NA | NA | 86 | 100 |
| (Matos et al., 2011) | Applicability of an optimized non-conventional flow cytometry method to detect anti-Trypanosoma cruzi immunoglobulin G for the serological diagnosis and cure assessment following chemotherapeutic treatment of Chagas disease | Matos et al. In-house Flow Cytometry | Flow Cytometry | NA | NA | NA | 98.1 | 100 |
| (Mendicino et al., 2019) | Simultaneous use of two rapid diagnostic tests for the diagnosis of Chagas disease. | WL Check Chagas | RDT | Wiener Laboratorios S.A.I.C. | EU/Argentina | Yes | 90.5 | 100 |
|  |  | SD BIOLINE Chagas Ab Rapid | RDT | Standard Diagnostics, Inc. (Now Abbott Laboratories) | EU | Yes | 97.6 | 93.8 |
| (Mita-Mendoza et al., 2018) | Chagas Disease in Southern Coastal Ecuador: Coinfections with Arboviruses and a Comparison of Serological Assays for Chagas Disease Diagnosis | ORTHO *T. cruzi* ELISA Test System | ELISA | Ortho-Clinical Diagnostics | FDA/EU | Yes | NA | NA |
|  |  | Hemagen Chagas’ Kit | ELISA | Hemagen Diagnostics, Inc. | EU | Yes | NA | NA |
|  |  | Chagatest ELISA recombinate v.3.0 | ELISA | Wiener Laboratorios S.A.I.C. | FDA | Yes | NA | NA |
|  |  | Chagas Detect Plus | RDT | InBios International, Inc. | FDA | Yes | NA | NA |
|  |  | Mita-Mendoza et al. 2018 TESAblot | Western-Blot | NA | NA | NA | NA | NA |
| (Moser et al., 2023a) | Concordance of Results by Three Chagas Disease Antibody Assays in U.S. Clinical Specimens. | Wiener Chagatest ELISA v3.0 | ELISA |  |  |  |  |  |
|  |  | Hemagen Chagas Kit ELISA |  |  |  |  |  |  |
|  |  | InBios Chagas Detect Fast Elisa |  |  |  |  |  |  |
| (Moser et al., 2023b) | Evaluation of InBios Chagas Detect Fast, a Novel Enzyme-Linked Immunosorbent Assay for the Detection of Anti-Trypanosoma cruzi Antibodies. | Chagas Detect Fast ELISA | ELISA | InBios International, Inc. | NA | Yes | 98.7 | 98.2 |
| (Mucci et al., 2017) | Next-generation ELISA diagnostic assay for Chagas Disease based on the combination of short peptidic epitopes. | Mucci et al. 2017 In-house ELISA | ELISA | NA | NA | NA | 96.3 | 99.15 |
| (Oelemann et al., 1998) | Evaluation of three commercial enzyme-linked immunosorbent assays for diagnosis of Chagas' disease | Abbott Chagas antibody EIA | ELISA | Abbott | NA | No | 99.0 | 96.8 |
|  |  | Bioelisa Chagas | ELISA | Werfen, S.A. | EU | Yes | 98.6 | 99.8 |
|  |  | Biozima Chagas | ELISA | Laboratórios Lemos S.R.L. | Argentina | Yes | 100 | 94.7 |
| (Otani et al., 2009) | WHO comparative evaluation of serologic assays for Chagas disease | HBK 401 Hemobio Chagas | ELISA | Embrabio | NA | No | 100 | 99.62 |
|  |  | Chagas ELISA | ELISA | Ebram | NA | No | 97.62 | 97.71 |
|  |  | Chagatek ELISA | ELISA | Laboratórios Lemos S.R.L. | Argentina | Yes | 99.4 | 99.24 |
|  |  | Premier Chagas IgG ELISA Test | ELISA | Meridian Diagnostics | NA | No | 94.04 | 100 |
|  |  | Test ELISA para Chagas | ELISA | BiosChile | NA | No | 99.4 | 99.62 |
|  |  | Bioelisacruzi | ELISA | Biolab-Mérieux, S.A. | NA | No | 98.21 | 99.24 |
|  |  | Abbott Chagas Anticorpos EIA | ELISA | Abbott | NA | No | 99.4 | 98.09 |
|  |  | Chagas test IICS, ELISA | ELISA | IICS Univ de Asuncion | NA | No | 97.02 | 99.24 |
|  |  | Chagatest ELISA recombinate v.3.0 | ELISA | Wiener Laboratorios S.A.I.C. | FDA | Yes | 98.81 | 99.62 |
|  |  | Bioelisa Chagas | ELISA | Werfen, S.A. | EU | Yes | 100 | 99.24 |
|  |  | Hemagen Chagas’ Kit | ELISA | Hemagen Diagnostics, Inc. | EU | Yes | 100 | 96.56 |
|  |  | Chagas HAI Imunoserum (Now HAI Chagas Polychaco) | IHA | Laboratórios Lemos S.R.L. | Argentina | Yes | 97.62 | 78.62 |
|  |  | Teste Chagas-HAI | IHA | Ebram | NA | No | 88.09 | 59.92 |
|  |  | Imuno-HAI Chagas | IHA | Wama Diagnóstica | NA | Yes | 100 | 95.8 |
|  |  | Chagas Hemagen HA | IHA | Hemagen Diagnostics, Inc. | NA | No | 92.26 | 89.31 |
|  |  | Hemacruzi | IHA | Biolab-Mérieux, S.A. | NA | No | 99.4 | 97.33 |
|  |  | Serodia Chagas | RDT | Fujirebio, Inc. | NA | No | 100 | 97.7 |
|  |  | Chagas STAT PAK Assay | RDT | Chembio Diagnostic Systems, Inc. | EU/Argentina | Yes | 94.08 | 99.62 |
| (Pereira et al., 2012) | Performance of six diagnostic tests to screen for Chagas disease in blood banks andprevalence of Trypanosoma cruzi infection among donors with inconclusive serologyscreening based on the analysis of epidemiological variables | Pereira et al. In-house Blood Culture Test | Blood Culture | NA | NA | NA | 58.07 | 100 |
|  |  | Chagatest ELISA recombinate v.3.0 | ELISA | Wiener Laboratorios S.A.I.C. | FDA | Yes | 97.76 | 97.25 |
|  |  | Pereira et al. In-house c-ELISA (using Bio-Manguinhos kit) | ELISA | Bio-Manguinhos | NA | No | 97.63 | 97.71 |
|  |  | Pereira et al. In-house rec-ELISA (using Bio-Manguinhos kit) | ELISA | Bio-Manguinhos | NA | No | 97.76 | 97.77 |
|  |  | Chagatest IHA | IHA | Wiener Laboratorios S.A.I.C. | EU/Argentina | Yes | 97.7 | 98.48 |
|  |  | IMUNOCRUZI | IIF | Biolab-Mérieux, S.A. | Brazil | No | 97.64 | 98.52 |
| (Pérez-Ayala et al., 2018) | Usefulness of the ARCHITECT Chagas(¬Æ) assay as a single test for the diagnosis of chronic Chagas disease. | ARCHITECT Chagas assay | CMIA | Abbott | EU/Brazil | No | 92.5 | 100 |
| (Peverengo et al., 2021) | Congenital chagas disease: Development and assessment of a specific IgM capture-based assay for diagnosis of transmission. | Peverengo et al. In-house ELISA Capture IgM | ELISA | NA | NA | NA | 80.75 | 93.1 |
| (Ponce et al., 2005) | Validation of a rapid and reliable test for diagnosis of chagas' disease by detection of Trypanosoma cruzi-specific antibodies in blood of donors and patients in Central America | Chagas STAT PAK Assay | RDT | Chembio Diagnostic Systems, Inc. | EU/Argentina | Yes | 99.6 | 99.9 |
| (Praast et al., 2011) | Evaluation of the Abbott ARCHITECT Chagas prototype assay | ARCHITECT Chagas assay | CMIA | Abbott | EU/Brazil | No | 99.85 | 99.99 |
| (Ramírez et al., 2009) | Evaluation of Adult Chronic Chagas' Heart Disease Diagnosis by Molecular and Serological Methods | Ramírez et al. In-house stDNA PCR | qPCR | NA | NA | NA | 75 | 100 |
|  |  | Ramírez et al. In-house stDNA PCR | qPCR | NA | NA | NA | 70 | 100 |
| (Ramírez et al., 2018) | Evaluation of the analytical and diagnostic performance of a digital droplet polymerase chain reaction (ddPCR) assay to detect Trypanosoma cruzi DNA in blood samples | Ramírez et al. In-house digital droplet (dd)PCR | ddPCR | NA | NA | NA | 100 | 100 |
| (Reiche et al., 1998) | Evaluation of the western blot in the confirmatory serologic diagnosis of Chagas' disease | Reiche et al. 1998 In-house Western-Blot | Western-Blot | NA | NA | NA | 86.6 | 100 |
| (Reis-Cunha et al., 2014) | Genome-wide screening and identification of new Trypanosoma cruzi antigens with potential application for chronic Chagas disease diagnosis. | Reis-Cunha et al. 2014 In-house ELISA (with rTc_11623.20 and rTc_N_10421.310 proteins) | ELISA | NA | NA | NA | 96.55 | 98.18 |
| (Reithinger et al., 2010) | Rapid detection of *Trypanosoma cruzi* in human serum by use of an immunochromatographic dipstick test | Trypanosoma Detect MRA rapid test (Replaced by Chagas Detect) | RDT | InBios International, Inc. | NA | No | 84.8 | 97.9 |
| (Rivera et al., 2022) | Evaluation of the Performance of Ortho *T. cruzi* ELISA Test System for the Detection of Antibodies to *Trypanosoma cruzi.* | ORTHO *T. cruzi* ELISA Test System | ELISA | Ortho-Clinical Diagnostics | FDA/EU | Yes |  |  |
| (Sánchez-Camargo et al., 2014) | Comparative evaluation of 11 commercialized rapid diagnostic tests for detecting Trypanosoma cruzi antibodies in serum banks in areas of endemicity and nonendemicity | Chagas Ab Combo Rapid Test CE | RDT | CTK Biotech, Inc. | EU | Yes | 90.1 | 91 |
|  |  | WL Check Chagas | RDT | Wiener Laboratorios S.A.I.C. | EU/Argentina | Yes | 88.7 | 97 |
|  |  | Chagas Instantest | RDT | Silanes | NA | No | 76.6 | 79 |
|  |  | Trypanosoma Detect MRA rapid test (Replaced by Chagas Detect) | RDT | InBios International, Inc. | NA | No | 92.9 | 94 |
|  |  | Cypress Chagas Quick Test | RDT | Cypress Diagnostics | EU | No | 92.9 | 93.2 |
|  |  | Chagas STAT PAK Assay | RDT | Chembio Diagnostic Systems, Inc. | EU/Argentina | Yes | 87.2 | 93.2 |
|  |  | Immu-Sure Chagas (*T. Cruzi*) | RDT | Millennium Biotech | NA | No | 10.6 | 97 |
|  |  | SD BIOLINE Chagas Ab Rapid | RDT | Standard Diagnostics, Inc. (Now Abbott Laboratories) | EU | Yes | 90.7 | 94 |
|  |  | Operon immunochromatographic test (ICT-Operon Simple Chagas WB [whole blood]) | RDT | Operon, S.A. | NA | No | 84.9 | 70.7 |
|  |  | Serodia Chagas | RDT | Fujirebio, Inc. | NA | No | 94.2 | 94.7 |
|  |  | ImmunoComb II Chagas Ab | RDT | Orgenics | NA | No | 97.2 | 94 |
| (Santos et al., 2016) | Chronic Chagas Disease Diagnosis: A Comparative Performance of Commercial Enzyme Immunoassay Tests | ELISA Chagas III | ELISA | BiosChile | NA | No | 97.3 | 100 |
|  |  | Imuno-ELISA Chagas | ELISA | Wama Diagnóstica | NA | No | 99.5 | 99.2 |
|  |  | Gold ELISA Chagas | ELISA | REM | NA | No | 100 | 100 |
|  |  | Pathozyme Chagas | ELISA | Omega Diagnostics Ltd | NA | No | 99.5 | 97 |
| (Santos et al., 2018) | Highly Accurate Chimeric Proteins for the Serological Diagnosis of Chronic Chagas Disease: A Latent Class Analysis | Santos et al.2018 In-house ELISA | ELISA | NA | NA | NA | 96.525 | 99.8 |
| (Santos et al., 2022) | Performance of Chimeric *Trypanosoma cruzi* Antigens in Serological Screening for Chagas Disease in Blood Banks. | Santos et al.2022 In-house ELISA | ELISA | NA | NA | NA | 92.86 | 99.99 |
| (Santos et al., 2021) | Assessment of Liaison XL Murex Chagas diagnostic performance in blood screening for Chagas disease using a reference array of chimeric antigens. | Liaison XL Murex Chagas CILA | CMIA | DiaSorin S.p.A. | EU | Yes | 76.2 | 99.5 |
| (Schaumburg et al., 2023) | Coupling ELISA to smartphones for POCT of chronic and congenital Chagas disease. | Schaumburg et al. In-house ELISA-based POCT with Smartphone app | ELISA | NA | NA | NA | 100 | 99.3 |
| (Schijman et al., 2011) | International study to evaluate PCR methods for detection of Trypanosoma cruzi DNA in blood samples from Chagas disease patients | Schijman et al. In-house cPCR | cPCR | NA | NA | NA | 55.9 | 68.8 |
|  |  | Schijman et al. In-house qPCR | qPCR | NA | NA | NA | 68.4 | 77.5 |
| (Shah et al., 2014) | Field evaluation of the InBios Chagas detect plus rapid test in serum and whole-blood specimens in Bolivia. | Chagas Detect Plus | RDT | InBios International, Inc. | FDA | Yes | 97.75 | 97.85 |
| (Silgado et al., 2021) | Analytical Evaluation of Dried Blood Spot and Rapid Diagnostic Test as a New Strategy for Serological Community Screening for Chronic Chagas Disease. | Elecsys Chagas (E-CILA: Electrochemiluminiscence)** | CMIA | Roche Diagnostics | EU | Yes | 96.9 | 100 |
|  |  | Chagas ELISA IgG + IgM | ELISA | Vircell S.L. | EU | Yes | 77.4 | 100 |
|  |  | Trypanosoma Detect MRA rapid test (Replaced by Chagas Detect) | RDT | InBios International, Inc. | NA | No | 89.6 | 100 |
| (Tobler et al., 2007) | Evaluation of a new enzyme-linked immunosorbent assay for detection of Chagas antibody in US blood donors | ORTHO *T. cruzi* ELISA Test System | ELISA | Ortho-Clinical Diagnostics | FDA/EU | Yes | 97.7 | 100 |
| (Torcoroma-García et al., 2021) | Disagreement between PCR and serological diagnosis of Trypanosoma cruzi infection in blood donors from a Colombian endemic region | ARCHITECT Chagas assay | CMIA | Abbott | EU/Brazil | No | 16.7 | 100 |
| (Valdez et al., 2016) | Diagnosis of Congenital Chagas Disease Using an Iron Superoxide Dismutase Excreted as Antigen, in Mothers and Their Children During the First Year of Life | Valdez et al. In-house ELISA/WB | ELISA/Western-Blot | NA | NA | NA | 100 | 99.32 |
| (Virreira et al., 2003) | Comparison of polymerase chain reaction methods for reliable and easy detection of congenital Trypanosoma cruzi infection | 3 different PCR systems (FastStart, Platinium, and Accuprime) with different antigens and in-house primers | cPCR | NA | NA | NA | NA | NA |
| (Wehrendt et al., 2021) | Development and Evaluation of a Three-Dimensional Printer-Based DNA Extraction Method Coupled to Loop Mediated Isothermal Amplification for Point-of-Care Diagnosis of Congenital Chagas Disease in Endemic Regions | Trypanosoma cruzi Loopamp prototype kit | LAMP | Eiken Chemical Company | NA | No | 100 | 100 |
| (Whitman et al., 2019) | Chagas Disease Serological Test Performance in U.S. Blood Donor Specimens. | Hemagen Chagas’ Kit | ELISA | Hemagen Diagnostics, Inc. | EU | Yes | 88 | 100 |
|  |  | ORTHO *T. cruzi* ELISA Test System | ELISA | Ortho-Clinical Diagnostics | FDA/EU | Yes | 92.4 | 100 |
|  |  | Chagatest ELISA recombinate v.3.0 | ELISA | Wiener Laboratorios S.A.I.C. | FDA | Yes | 94 | 99.33 |
|  |  | Chagas Detect Plus | RDT | InBios International, Inc. | FDA | Yes | 97.4 | 92.33 |

CMIA: Chemiluminiscence, cPCR: Conventional PCR, qPCR: Real-time or Quantitative PCR, ddPCR: Digital droplet PCR, ELISA: Enzyme-Linked Immunosorbent Assay, EU: European Union, FDA: Food and Drug Administration, IIF: Immunofluorescence, IHA: Indirect hemagglutination, LAMP: Loop-mediated isothermal amplification, NA: Not apply, ND: No data, RDT: Rapid Immunochromatographic test, RIPA: Radioimmunoprecipitation assay.

Abras, A., Ballart, C., Llovet, T., Roig, C., Gutiérrez, C., Tebar, S., Berenguer, P., Pinazo, M.J., Posada, E., Gascón, J., Schijman, A.G., Gállego, M., and Muñoz, C. (2018). "Introducing automation to the molecular diagnosis of Trypanosoma cruzi infection: A comparative study of sample treatments, DNA extraction methods and real-time PCR assays", in: *PLoS ONE.*).

Abras, A., Gállego, M., Llovet, T., Tebar, S., Herrero, M., Berenguer, P., Ballart, C., Martí, C., and Muñoz, C. (2016). "Serological diagnosis of chronic chagas disease: Is it time for a change?", in: *Journal of Clinical Microbiology.*).

Almeida, I.C., Covas, D.T., Soussumi, L.M.T., and Travassos, L.R. (1997). "A highly sensitive and specific chemiluminescent enzyme-linked immunosorbent assay for diagnosis of active Trypanosoma cruzi infection", in: *Transfusion.*).

Angheben, A., Staffolani, S., Anselmi, M., Tais, S., Degani, M., Gobbi, F., Buonfrate, D., Gobbo, M., and Bisoffi, Z. (2017). "Accuracy of a rapid diagnostic test (Cypress Chagas Quick Test®) for the diagnosis of chronic Chagas disease in a nonendemic area: A retrospective longitudinal study", in: *American Journal of Tropical Medicine and Hygiene.*).

Araújo, A.B., and Berne, M.E.A. (2013). "Conventional serological performance in diagnosis of Chagas’ disease in southern Brazil", in: *The Brazilian Journal of Infectious Diseases.*).

Azogue, E., and Darras, C. (1995). "Congenital Chagas in Bolivia: comparative study of the effectiveness and cost of diagnostic methods", in: *Revista da Sociedade Brasileira de Medicina Tropical.*).

Barfield, C.A., Barney, R.S., Crudder, C.H., Wilmoth, J.L., Stevens, D.S., Mora-Garcia, S., Yanovsky, M.J., Weigl, B.H., and Yanovsky, J. (2011). "A highly sensitive rapid diagnostic test for Chagas disease that utilizes a recombinant Trypanosoma cruzi antigen", in: *IEEE Transactions on Biomedical Engineering.*).

Besuschio, S.A., Llano Murcia, M., Benatar, A.F., Monnerat, S., Cruz Mata, I., Picado De Puig, A., Curto, M.D.L.Á., Kubota, Y., Wehrendt, D.P., Pavia, P., Mori, Y., Puerta, C., Ndung'u, J.M., Schijman, A.G., Cruz, I., Picado, A., Curto, M.D.L.Á., Kubota, Y., Wehrendt, D.P., Pavia, P., Mori, Y., Puerta, C., Ndung'u, J.M., and Schijman, A.G. (2017). "Analytical sensitivity and specificity of a loop-mediated isothermal amplification (LAMP) kit prototype for detection of Trypanosoma cruzi DNA in human blood samples", in: *PLoS Neglected Tropical Diseases.*).

Besuschio, S.A., Picado, A., Muñoz-Calderón, A., Wehrendt, D.P., Fernández, M., Benatarid, A., Diaz-Bello, Z., Irurtia, C., Cruz, I., Ndung’u, J.M., Cafferata, M.L., Montenegro, G., Estani, S.S., Lucero, R.H., De Noya, B.A., Longhi, S.A., and Schijman, A.G. (2020). "Trypanosoma cruzi loop-mediated isothermal amplification (Trypanosoma cruzi loopamp) kit for detection of congenital, acute and chagas disease reactivation", in: *PLoS Neglected Tropical Diseases.*).

Bisio, M.M.C., Rivero, R., Gonzalez, N., Ballering, G., D’amico, I., Kessler, C., Moroni, S., Moscatelli, G., Ruiz, A.M., and Altcheh, J. (2021). "Diagnostic Accuracy of Two Molecular Tools for Diagnosis of Congenital Chagas Disease", in: *Molecular Diagnosis and Therapy.*).

Brashear, R.J., Winkler, M.A., Schur, J.D., Lee, H., Burczak, J.D., Hall, H.J., and Pan, A.A. (1995). "Detection of antibodies to Trypanosoma cruzi among blood donors in the southwestern and western United States. I. Evaluation of the sensitivity and specificity of an enzyme immunoassay for detecting antibodies to T. cruzi", in: *Transfusion.*).

Brossas, J.Y., Griselda, B., Bisio, M., Guihenneuc, J., Gulin, J.E.N., Jauréguiberry, S., Lescure, F.X., Fekkar, A., Mazier, D., Altcheh, J., and Paris, L. (2021). "Evaluation of the chagas western blot igg assay for the diagnosis of chagas disease", in: *Pathogens.*).

Carvalho, M.R., Krieger, M.A., Almeida, E., Oelemann, W., Shikanai‐Yassuda, M.A., Ferreira, A.W., Pereira, J.B., Saez‐Alquezar, A., Dorlhiac‐Llacer, P.E., Chamone, D.F., and Goldenberg, S. (1993). "Chagas' disease diagnosis: evaluation of several tests in blood bank screening", in: *Transfusion.*).

Castro-Sesquen, Y.E., Saldaña, A., Patino Nava, D., Bayangos, T., Paulette Evans, D., Detoy, K., Trevino, A., Marcus, R., Bern, C., Gilman, R.H., and Talaat, K.R. (2021a). "Use of a Latent Class Analysis in the Diagnosis of Chronic Chagas Disease in the Washington Metropolitan Area", in: *Clinical Infectious Diseases.*).

Castro-Sesquen, Y.E., Saldaña, A., Patino Nava, D., Paulette Evans, D., Bayangos, T., Detoy, K., Trevino, A., Marcus, R., Bern, C., Gilman, R.H., Talaat, K.R., Avila, C., Camacho, F., Herrera, S., Jimenez, A., Lozano, V., Malaga, E., Merida, M., Morales, C., Solis, R., Sotomayor, F., Tung, A., Spector, A., Verastegui, M., Yang, Y., and Zapata, F. (2021b). "Evaluation of 2 lateral flow rapid tests in the diagnosis of chagas disease in the washington metropolitan area", in: *Open Forum Infectious Diseases.*).

Castro-Sesquen, Y.E., Tinajeros, F., Bern, C., Galdos-Cardenas, G., Malaga, E.S., Valencia Ayala, E., Hjerrild, K., Clipman, S.J., Lescano, A.G., Bayangos, T., Castillo, W., Menduiña, M.C., Talaat, K.R., Gilman, R.H., Verastegui, M., Calderon, M., Chávez, C., Leigue, J.K., Hinojosa, E., Urquizu, F., Gorena, M., Serrudo, V., Cabrera, L., and Romero, Y.K. (2021c). "The immunoglobulin M-Shed acute phase antigen (SAPA)-test for the early diagnosis of congenital Chagas disease in the time of the elimination goal of mother-to-child transmission", in: *Clinical Infectious Diseases.*).

Chappuis, F., Mauris, A., Holst, M., Albajar-Vinas, P., Jannin, J., Luquetti, A.O., and Jackson, Y. (2010). "Validation of a rapid immunochromatographic assay for diagnosis of Trypanosoma cruzi infection among Latin-American migrants in Geneva, Switzerland", in: *Journal of Clinical Microbiology.*).

Cortes-Serra, N., Pinazo, M.J., De La Torre, L., Galizzi, M., Gascon, J., and Bustamante, J.M. (2018). "Diagnosis of trypanosoma cruzi infection status using saliva of infected subjects", in: *American Journal of Tropical Medicine and Hygiene.*).

Daltro, R.T., Santos, E.F., Silva, Â.a.O., Freitas, N.E.M., Leony, L.M., Vasconcelos, L.C.M., Luquetti, A.O., Celedon, P.a.F., Zanchin, N.I.T., Regis-Silva, C.G., and Santos, F.L.N. (2022). "Western blot using Trypanosoma cruzi chimeric recombinant proteins for the serodiagnosis of chronic Chagas disease: A proof-of-concept study", in: *PLoS neglected tropical diseases.* PLoS Negl Trop Dis).

De Oliveira, L.C., Pereira, N.B., Moreira, C.H.V., Bierrenbach, A.L., Salles, F.C., De Souza-Basqueira, M., Manuli, E.R., Ferreira, A.M., Di Lorenzo Oliveira, C., Cardoso, C.S., Ribeiro, A.L.P., and Sabino, E.C. (2020). "ELISA Saliva for Trypanosoma cruzi Antibody Detection: An alternative for serological surveys in endemic regions", in: *American Journal of Tropical Medicine and Hygiene.*).

Duarte, L.F., Flórez, O., Rincón, G., and González, C.I. (2014). "Comparison of seven diagnostic tests to detect Trypanosoma cruzi infection in patients in chronic phase of Chagas disease", in: *Colombia Medica.*).

Egüez, K.E., Alonso-Padilla, J., Terán, C., Chipana, Z., García, W., Torrico, F., Gascon, J., Lozano-Beltran, D.F., and Pinazo, M.J. (2017). "Rapid diagnostic tests duo as alternative to conventional serological assays for conclusive Chagas disease diagnosis", in: *PLoS Neglected Tropical Diseases.*).

Feilij, H., Muller, L., and Gonzalez Cappa, S.M. (1983). "Direct micromethod for diagnosis of acute and congenital Chagas' disease", in: *Journal of Clinical Microbiology.*).

Ferreira, A.W., Belem, Z.R., Lemos, E.A., Reed, S.G., and Campos-Neto, A. (2001). "Enzyme-linked immunosorbent assay for serological diagnosis of chagas' disease employing a Trypanosoma cruzi recombinant antigen that consists of four different peptides", in: *Journal of Clinical Microbiology.*).

Ferreira-Silva, M.M., Pereira, G.D.A., Rodrigues-Júnior, V., Meira, W.S., Basques, F.V., Langhi-Júnior, D.M., Romanelli, M., Umezawa, E.S., Késper-Júnior, N., Louzada-Neto, F., Bordin, J.O., and Moraes-Souza, H. (2021). "Chagas disease: Performance analysis of immunodiagnostic tests anti-Trypanosoma cruzi in blood donors with inconclusive screening results", in: *Hematology, Transfusion and Cell Therapy.*).

Flores-Chavez, M., Cruz, I., Nieto, J., Gárate, T., Navarro, M., Pérez-Ayala, A., López-Vélez, R., and Cañavate, C. (2012). "Sensitivity and specificity of an operon immunochromatographic test in serum and whole-blood samples for the diagnosis of Trypanosoma cruzi infection in Spain, an area of nonendemicity", in: *Clinical and Vaccine Immunology.*).

Flores-Chavez, M.D., Abras, A., Ballart, C., Perez, I.I., Perez-Gordillo, P., Gállego, M., Muñoz, C., Moure, Z., Igual, E.S., Nieto, J., Diez, E.G., Cruz, I., and Picado, A. (2021). "Evaluation of the performance of the loopamp trypanosoma cruzi detection kit for the diagnosis of chagas disease in an area where it is not endemic, Spain", in: *Journal of Clinical Microbiology.*).

Flores-Chavez, M.D., Sambri, V., Schottstedt, V., Higuera-Escalante, F.A., Roessler, D., Chaves, M., Laengin, T., Martinez, A., and Fleischer, B. (2018). "Evaluation of the elecsys chagas assay for detection of trypanosoma cruzi-specific antibodies in a multicenter study in Europe and Latin America", in: *Journal of Clinical Microbiology.*).

Frade, A.F., Luquetti, A.O., Prata, A., and Ferreira, A.W. (2011). "Western blotting method (TESAcruzi) as a supplemental test for confirming the presence of anti-Trypanosoma cruzi antibodies in finger prick blood samples from children aged 0-5 years in Brazil", in: *Acta Tropica.*).

Gamboa-León, R., Gonzalez-Ramirez, C., Padilla-Raygoza, N., Sosa-Estani, S., Caamal-Kantun, A., Buekens, P., and Dumonteil, E. (2011). "Do commercial serologic tests for trypanosoma cruzi infection detect mexican strains in women and newborns?", in: *Journal of Parasitology.*).

García-Bermejo, I., Arana, D.M., Zaragoza Vargas, G., Carrasco Fernández, B., García, E., Nieto, J., and Flores-Chávez, M.D. (2022). "Evaluation of the Chagas VirClia® and Chagas TESA VirClia® for the Diagnosis of Trypanosoma cruzi Infection", in: *Pathogens (Basel, Switzerland).* Pathogens).

Gil-Gallardo, L., Simón, M., Iborra, A., Carrilero, B., and Segovia, M. (2021). "Chemiluminescent Microparticle Immunoassay for the Diagnosis of Congenital Chagas Disease: A Prospective Study in Spain", in: *The American Journal of Tropical Medicine and Hygiene.*).

Gomes, M.L., Galvao, L.M.C., Macedo, A.M., Pena, S.D.J., and Chiari, E. (1999). "Chagas' disease diagnosis: Comparative analysis of parasitologic, molecular, and serologic methods", in: *American Journal of Tropical Medicine and Hygiene.*).

Gorlin, J., Rossmann, S., Robertson, G., Stallone, F., Hirschler, N., Nguyen, K.A., Gilcher, R., Fernandes, H., Alvey, S., Ajongwen, P., Contestable, P., and Warren, H. (2008). "Evaluation of a new Trypanosoma cruzi antibody assay for blood donor screening", in: *Transfusion.*).

Hamerschlak, N., Pasternak, J., Amato Neto, V., De Carvalho, M.B., Guerra, C.S., Coscina, A.L., Ferreira, O.C., Rosenblit, J., and Szterling, L.N. (1997). "Chagas' disease: an algorithm for donor screening and positive donor counseling", in: *Revista da Sociedade Brasileira de Medicina Tropical.* Rev Soc Bras Med Trop).

Hernández, C., Cucunubá, Z., Flórez, C., Olivera, M., Valencia, C., Zambrano, P., León, C., and Ramírez, J.D. (2016). "Molecular Diagnosis of Chagas Disease in Colombia: Parasitic Loads and Discrete Typing Units in Patients from Acute and Chronic Phases", in: *PLOS Neglected Tropical Diseases.*).

Hernández, C., Teherán, A., Flórez, C., and Ramírez, J.D. (2018). "Comparison of parasite loads in serum and blood samples from patients in acute and chronic phases of Chagas disease", in: *Parasitology.*).

Hernández, I., Hernández, M., González, J., Gómez, I., Zulueta, O., Ramos, G., Ortega, D., Bequer, D.C., Martínez, G.E., and Delahanty, A. (2021). "Evaluation of UMELISA CHAGAS™ with the incorporation of new monomeric and chimeric peptides representative of different regions of Trypanosoma cruzi", in: *Biomedica.*).

Holguín, A., Norman, F., Martín, L., Mateos, M.L., Chacón, J., López-Vélez, R., and Pérez-Molina, J.A. (2013). "Dried blood as an alternative to plasma or serum for Trypanosoma cruzi IgG detection in screening programs", in: *Clinical and Vaccine Immunology.*).

Iturra, J.a.D., Leony, L.M., Medeiros, F.a.C., Souza Filho, J.a.D., Siriano, L.D.R., Tavares, S.B., Luquetti, A.O., Belo, V.S., Sousa, A.S.D., and Santos, F.L.N. (2023). "A multicenter comparative study of the performance of four rapid immunochromatographic tests for the detection of anti- Trypanosoma cruzi antibodies in Brazil", in: *Frontiers in medicine.* Front Med (Lausanne)).

Junqueira, A.C.V., Chiari, E., and Wincker, P. (1996). "Comparison of the polymerase chain reaction with two classical parasitological methods for the diagnosis of Chagas disease in an endemic region of north-eastern Brazil", in: *Transactions of the Royal Society of Tropical Medicine and Hygiene.*).

Kann, S., Kunz, M., Hansen, J., Sievertsen, J., Crespo, J.J., Loperena, A., Arriens, S., and Dandekar, T. (2020). "Chagas disease: Detection of trypanosoma cruzi by a new, high-specific real time PCR", in: *Journal of Clinical Medicine.*).

Kelly, E.A., Bulman, C.A., Gunderson, E.L., Irish, A.M., Townsend, R.L., Sakanari, J.A., Stramer, S.L., Bern, C., and Whitman, J.D. (2021). "Comparative performance of latest-generation and FDA-cleared serology tests for the diagnosis of chagas disease", in: *Journal of Clinical Microbiology.*).

Kim, Y.H., Yang, Z., Lee, J., Ahn, H.J., Chong, C.K., Maricondi, W., Dias, R.F., and Nam, H.W. (2019). "Detection of human anti-trypanosoma cruzi antibody with recombinant fragmented ribosomal P protein", in: *Korean Journal of Parasitology.*).

Leiby, D.A., Wendel, S., Takaoka, D.T., Fachini, R.M., Oliveira, L.C., and Tibbals, M.A. (2000). "Serologic testing for Trypanosoma cruzi: comparison of radioimmunoprecipitation assay with commercially available indirect immunofluorescence assay, indirect hemagglutination assay, and enzyme-linked immunosorbent assay kits", in: *Journal of clinical microbiology.* J Clin Microbiol).

Llano, M., Pavía, P., Flórez, A.C., Cuéllar, A., González, J.M., and Puerta, C. (2014). "Preliminary evaluation of the commercial kit Chagas (Trypanosoma cruzi) IgG-ELISA® in colombian individuals | Evaluación preliminar de la prueba comercial Chagas (Trypanosoma cruzi) IgG-ELISA® en individuos colombianos", in: *Biomedica.*).

Longhi, S.A., Brandariz, S.B., Lafon, S.O., Niborski, L.L., Luquetti, A.O., Schijman, A.G., Levin, M.J., and Gómez, K.A. (2012). "Evaluation of in-house ELISA using Trypanosoma cruzi lysate and recombinant antigens for diagnosis of chagas disease and discrimination of its clinical forms", in: *American Journal of Tropical Medicine and Hygiene.*).

Longhi, S.A., García Casares, L.J., Muñoz-Calderón, A.A., Alonso-Padilla, J., and Schijman, A.G. (2023). "Combination of ultra-rapid DNA purification (PURE) and loop-mediated isothermal amplification (LAMP) for rapid detection of Trypanosoma cruzi DNA in dried blood spots", in: *PLOS Neglected Tropical Diseases.*).

Lozano, D., Rojas, L., Méndez, S., Casellas, A., Sanz, S., Ortiz, L., Pinazo, M.J., Abril, M., Gascón, J., Torrico, F., and Alonso-Padilla, J. (2019). "Use of rapid diagnostic tests (RDTs) for conclusive diagnosis of chronic Chagas disease - Field implementation in the Bolivian Chaco region", in: *PLoS Neglected Tropical Diseases.*).

Machado, J.M., Pereira, I.a.G., Maia, A.C.G., Francisco, M.F.C., Nogueira, L.M., Gandra, I.B., Ribeiro, A.J., Silva, K.A., Resende, C.a.A., Da Silva, J.O., Dos Santos, M., Gonçalves, A.a.M., Tavares, G.D.S.V., Chávez-Fumagalli, M.A., Campos-Da-Paz, M., Giunchetti, R.C., Rocha, M.O.D.C., Chaves, A.T., Coelho, E.a.F., and Galdino, A.S. (2023). "Proof of Concept of a Novel Multiepitope Recombinant Protein for the Serodiagnosis of Patients with Chagas Disease", in: *Pathogens (Basel, Switzerland).* Pathogens).

Marcon, G.E.B., Andrade, P.D., De Albuquerque, D.M., Da Wanderley, J.S., De Almeida, E.A., Guariento, M.E., and Costa, S.C.B. (2002). "Use of a nested polymerase chain reaction (N-PCR) to detect Trypanosoma cruzi in blood samples from chronic chagasic patients and patients with doubtful serologies", in: *Diagnostic Microbiology and Infectious Disease.*).

Matos, C.S., Coelho-Dos-Reis, J.G.A., Rassi, A., Luquetti, A.O., Dias, J.C.P., Eloi-Santos, S.M., Gomes, I.T., Vitelli-Avelar, D.M., Wendling, A.P.B., Rocha, R.D.R., Teixeira-Carvalho, A., Peruhype-Magalhães, V., Andrade, M.C., and Martins-Filho, O.A. (2011). "Applicability of an optimized non-conventional flow cytometry method to detect anti-Trypanosoma cruzi immunoglobulin G for the serological diagnosis and cure assessment following chemotherapeutic treatment of Chagas disease", in: *Journal of Immunological Methods.*).

Mendicino, D., Colussi, C., and Moretti, E. (2019). "Simultaneous use of two rapid diagnostic tests for the diagnosis of Chagas disease", in: *Tropical Doctor.*).

Mita-Mendoza, N.K., Mcmahon, E., Kenneson, A., Barbachano-Guerrero, A., Beltran-Ayala, E., Cueva, C., King, C.A., Lupone, C.D., Castro-Sesquen, Y.E., Gilman, R.H., Endy, T.P., and Stewart-Ibarra, A.M. (2018). "Chagas Disease in Southern Coastal Ecuador: Coinfections with Arboviruses and a Comparison of Serological Assays for Chagas Disease Diagnosis.", in: *The American journal of tropical medicine and hygiene.*).

Moser, M.S., Fleischmann, C.J., Kelly, E.A., Prince, H.E., Bern, C., and Whitman, J.D. (2023a). "Concordance of Results by Three Chagas Disease Antibody Assays in U.S. Clinical Specimens", in: *Journal of clinical microbiology.* J Clin Microbiol).

Moser, M.S., Fleischmann, C.J., Kelly, E.A., Townsend, R.L., Stramer, S.L., Bern, C., and Whitman, J.D. (2023b). "Evaluation of InBios Chagas Detect Fast, a Novel Enzyme-Linked Immunosorbent Assay for the Detection of Anti-Trypanosoma cruzi Antibodies", in: *Journal of clinical microbiology.* J Clin Microbiol).

Mucci, J., Carmona, S.J., Volcovich, R., Altcheh, J., Bracamonte, E., Marco, J.D., Nielsen, M., Buscaglia, C.A., and Agüero, F. (2017). "Next-generation ELISA diagnostic assay for Chagas Disease based on the combination of short peptidic epitopes", in: *PLoS Neglected Tropical Diseases.*).

Oelemann, W.M.R., Teixeira, M.D.G.M., Veríssimo Da Costa, G.C., Borges-Pereira, J., De Castro, J.a.F., Coura, J.R., and Peralta, J.M. (1998). "Evaluation of three commercial enzyme-linked immunosorbent assays for diagnosis of Chagas' disease", in: *Journal of Clinical Microbiology.*).

Otani, M.M., Vinelli, E., Kirchhoff, L.V., Del Pozo, A., Sands, A., Vercauteren, G., and Sabino, E.C. (2009). "WHO comparative evaluation of serologic assays for Chagas disease", in: *Transfusion.*).

Pereira, G.D.A., Louzada-Neto, F., Barbosa, V.D.F., Ferreira-Silva, M.M., and Moraes-Souza, H.D. (2012). "Performance of six diagnostic tests to screen for Chagas disease in blood banks and prevalence of Trypanosoma cruzi infection among donors with inconclusive serology screening based on the analysis of epidemiological variables", in: *Revista Brasileira de Hematologia e Hemoterapia.*).

Pérez-Ayala, A., Fradejas, I., Rebollo, L., Lora-Pablos, D., Lizasoain, M., and Herrero-Martínez, J.M. (2018). "Usefulness of the ARCHITECT Chagas® assay as a single test for the diagnosis of chronic Chagas disease", in: *Tropical Medicine and International Health.*).

Peverengo, L.M., Rodeles, L.M., Maldonado, C., Ballering, G., Pujato, N., D'amico, I., Vicco, M.H., Garcia, L., Jurado, L., Altcheh, J., and Marcipar, I. (2021). "Congenital chagas disease: Development and assessment of a specific IgM capture-based assay for diagnosis of transmission", in: *Acta Tropica.*).

Ponce, C., Ponce, E., Vinelli, E., Montoya, A., De Aguilar, V., Gonzalez, A., Zingales, B., Rangel-Aldao, R., Levin, M.J., Esfandiari, J., Umezawa, E.S., Luquetti, A.O., and Da Silveira, J.F. (2005). "Validation of a rapid and reliable test for diagnosis of Chagas' disease by detection of Trypanosoma cruzi-specific antibodies in blood of donors and patients in Central America", in: *Journal of Clinical Microbiology.*).

Praast, G., Herzogenrath, J., Bernhardt, S., Christ, H., and Sickinger, E. (2011). "Evaluation of the Abbott ARCHITECT Chagas prototype assay", in: *Diagnostic Microbiology and Infectious Disease.*).

RamíRez, J.D., Guhl, F., Umezawa, E.S., Morillo, C.A., Rosas, F., Marin-Neto, J.A., and Restrepo, S. (2009). "Evaluation of Adult Chronic Chagas' Heart Disease Diagnosis by Molecular and Serological Methods", in: *Journal of Clinical Microbiology.*).

Ramírez, J.D., Herrera, G., Hernández, C., Cruz-Saavedra, L., Muñoz, M., Flórez, C., and Butcher, R. (2018). "Evaluation of the analytical and diagnostic performance of a digital droplet polymerase chain reaction (ddPCR) assay to detect Trypanosoma cruzi DNA in blood samples", in: *PLOS Neglected Tropical Diseases.*).

Reiche, E.M.V., Cavazzana, M., Okamura, H., Tagata, E.C., Jankevicius, S.I., and Jankevicius, J.V. (1998). "Evaluation of the western blot in the confirmatory serologic diagnosis of Chagas' disease.", in: *The American Journal of Tropical Medicine and Hygiene.* American Society of Tropical Medicine and Hygiene).

Reis-Cunha, J.L., Mendes, T.a.D.O., De Almeida Lourdes, R., Ribeiro, D.R.D.S., Machado-De-Avila, R.A., De Oliveira Tavares, M., Lemos, D.S., Câmara, A.C.J., Olórtegui, C.C., De Lana, M., Galvão, L.M.D.C., Fujiwara, R.T., and Bartholomeu, D.C. (2014). "Genome-wide screening and identification of new Trypanosoma cruzi antigens with potential application for chronic chagas disease diagnosis", in: *PLoS ONE.*).

Reithinger, R., Grijalva, M.J., Chiriboga, R.F., De Noya, B.A., Torres, J.R., Pavia-Ruz, N., Manrique-Saide, P., Cardinal, M.V., and Gürtler, R.E. (2010). "Rapid detection of Trypanosoma cruzi in human serum by use of an immunochromatographic dipstick test", in: *Journal of Clinical Microbiology.*).

Rivera, H.N., Mcauliffe, I., Aderohunmu, T.L., Wiegand, R.E., Montgomery, S.P., Bradbury, R.S., and Handali, S. (2022). "Evaluation of the Performance of Ortho T. cruzi ELISA Test System for the Detection of Antibodies to Trypanosoma cruzi", in: *Journal of Clinical Microbiology.*).

Sánchez-Camargo, C.L., Albajar-Viñas, P., Wilkins, P.P., Nieto, J., Leiby, D.A., Paris, L., Scollo, K., Flórez, C., Guzmán-Bracho, C., Luquetti, A.O., Calvo, N., Tadokoro, K., Saez-Alquezar, A., Palma, P.P., Martin, M., and Flevaud, L. (2014). "Comparative evaluation of 11 commercialized rapid diagnostic tests for detecting Trypanosoma cruzi antibodies in serum banks in areas of endemicity and nonendemicity", in: *Journal of Clinical Microbiology.*).

Santos, E.F., Leony, L.M., Silva, Â.a.O., Daltro, R.T., Freitas, N.E.M., Vasconcelos, L.C.M., De Araújo, F.L.V., Celedon, P.a.F., Krieger, M.A., Zanchin, N.I.T., and Santos, F.L.N. (2021). "Assessment of Liaison XL Murex Chagas diagnostic performance in blood screening for Chagas disease using a reference array of chimeric antigens", in: *Transfusion.*).

Santos, E.F.D., Silva, A.a.O., Freitas, N.E.M., Leony, L.M., Daltro, R.T., Santos, C.a.D.S.T., Almeida, M.D.C.C.D., Araújo, F.L.V.D., Celedon, P.a.F., Krieger, M.A., Zanchin, N.I.T., Reis, M.G.D., and Santos, F.L.N. (2022). "Performance of Chimeric Trypanosoma cruzi Antigens in Serological Screening for Chagas Disease in Blood Banks", in: *Frontiers in Medicine.*).

Santos, F.L.N., Campos, A.C.P., Amorim, L.D.a.F., Silva, E.D., Zanchin, N.I.T., Celedon, P.a.F., Del-Rei, R.P., Krieger, M.A., and Gomes, Y.M. (2018). "Highly accurate chimeric proteins for the serological diagnosis of chronic chagas disease: A latent class analysis", in: *American Journal of Tropical Medicine and Hygiene.*).

Santos, F.L.N., De Souza, W.V., Da Silva Barros, M., Nakazawa, M., Krieger, M.A., and De Miranda Gomes, Y. (2016). "Chronic Chagas disease diagnosis: A comparative performance of commercial enzyme immunoassay tests", in: *American Journal of Tropical Medicine and Hygiene.*).

Schaumburg, F., Pujato, N., Peverengo, L.M., Marcipar, I.S., and Berli, C.L.A. (2023). "Coupling ELISA to smartphones for POCT of chronic and congenital Chagas disease", in: *Talanta.* Talanta).

Schijman, A.G., Bisio, M., Orellana, L., Sued, M., Duffy, T., Mejia Jaramillo, A.M., Cura, C., Auter, F., Veron, V., Qvarnstrom, Y., Deborggraeve, S., Hijar, G., Zulantay, I., Lucero, R.H., Velazquez, E., Tellez, T., Leon, Z.S., Galvão, L., Nolder, D., Rumi, M.M., Levi, J.E., Ramirez, J.D., Zorrilla, P., Flores, M., Jercic, M.I., Crisante, G., Añez, N., De Castro, A.M., Gonzalez, C.I., Viana, K.A., Yachelini, P., Torrico, F., Robello, C., Diosque, P., Chavez, O.T., Aznar, C., Russomando, G., Büscher, P., Assal, A., Guhl, F., Estani, S.S., Dasilva, A., Britto, C., Luquetti, A., and Ladzins, J. (2011). "International study to evaluate PCR methods for detection of Trypanosoma cruzi DNA in blood samples from Chagas disease patients", in: *PLoS Neglected Tropical Diseases.*).

Shah, V., Ferrufino, L., Gilman, R.H., Ramirez, M., Saenza, E., Malaga, E., Sanchez, G., Okamoto, E.E., Sherbuck, J.E., Clark, E.H., Galdos-Cardenas, G., Bozo, R., Flores-Franco, J.L., Colanzi, R., Verastegui, M., and Bern, C. (2014). "Field evaluation of the InBios Chagas detect plus rapid test in serum and whole-blood specimens in Bolivia", in: *Clinical and Vaccine Immunology.*).

Silgado, A., Gual-Gonzalez, L., Sánchez-Montalvá, A., Oliveira-Souto, I., Goterris, L., Serre-Delcor, N., Esperalba, J., Gomez-I-Prat, J., Fernández-Naval, C., Molina, I., Pumarola, T., and Sulleiro, E. (2021). "Analytical Evaluation of Dried Blood Spot and Rapid Diagnostic Test as a New Strategy for Serological Community Screening for Chronic Chagas Disease", in: *Frontiers in Cellular and Infection Microbiology.*).

Tobler, L.H., Contestable, P., Pitina, L., Groth, H., Shaffer, S., Blackburn, G.R., Warren, H., Lee, S.R., and Busch, M.P. (2007). "Evaluation of a new enzyme-linked immunosorbent assay for detection of Chagas antibody in US blood donors", in: *Transfusion.*).

Torcoroma-García, L., Aguilar, J.R., Bueno, M.Y., Moreno, E.M., Ramírez, H., and Daza, N. (2021). "Disagreement between PCR and serological diagnosis of Trypanosoma cruzi infection in blood donors from a Colombian endemic region", in: *Biomedica.*).

Valdez, F.C., Marín, C., Abuxapqui, J.F., Ortegón, J.E., Cañas, R., and Moreno, M.S. (2016). "Diagnosis of congenital chagas disease using an iron superoxide dismutase excreted as antigen, in mothers and their children during the first year of life", in: *Pediatric Infectious Disease Journal.*).

Virreira, M., Torrico, F., Truyens, C., Alonso-Vega, C., Solano, M., Carlier, Y., and Svoboda, M. (2003). "Comparison of polymerase chain reaction methods for reliable and easy detection of congenital Trypanosoma cruzi infection", in: *American Journal of Tropical Medicine and Hygiene.*).

Wehrendt, D.P., Alonso-Padilla, J., Liu, B., Rojas Panozo, L., Rivera Nina, S., Pinto, L., Lozano, D., Picado, A., Abril, M., Pinazo, M.J., Gascon, J., Torrico, F., Wong, S., and Schijman, A.G. (2021). "Development and Evaluation of a Three-Dimensional Printer–Based DNA Extraction Method Coupled to Loop Mediated Isothermal Amplification for Point-of-Care Diagnosis of Congenital Chagas Disease in Endemic Regions", in: *Journal of Molecular Diagnostics.*).

Whitman, J.D., Bulman, C.A., Gunderson, E.L., Irish, A.M., Townsend, R.L., Stramer, S.L., Sakanari, J.A., and Bern, C. (2019). "Chagas disease serological test performance in U.S. blood donor specimens", in: *Journal of Clinical Microbiology.*).
