# Supplementary material for: In vitro diagnostic methods of Chagas disease in the clinical laboratory: a scoping review
Source: Front Microbiol. 2024 Apr 30;15:1393992. doi: 10.3389/fmicb.2024.1393992 (PMC11091413; doi:10.3389/fmicb.2024.1393992)
Supplement: Supplementary file 3 [file Table_3.DOCX]

**Supplemental Table 3. Summary of discontinued serological tests**

| **Test** | **Method** | **Manufacturer** |
| --- | --- | --- |
| ELISA Chagas III | ELISA | BiosChile |
| Test ELISA para Chagas | ELISA | BiosChile |
| Imuno-ELISA Chagas | ELISA | Wama Diagnóstica |
| Gold ELISA Chagas | ELISA | REM |
| Pathozyme Chagas | ELISA | Omega Diagnostics Ltd |
| Biolab Diagnostica | ELISA | Biolab Diagnóstica |
| Gull Laboratories | ELISA | Gull Laboratories, Inc. |
| Abbott Chagas antibody EIA | ELISA | Abbott |
| DRG *Trypanosoma cruzi* IgG | ELISA | DRG International, Inc. |
| EIAgen T. cruzi IgG + IgM | ELISA | Adaltis |
| HBK 401 Hemobio Chagas | ELISA | Embrabio |
| Chagas ELISA | ELISA | Ebram |
| Premier Chagas IgG ELISA Test | ELISA | Meridian Diagnostics |
| Chagas test IICS, ELISA | ELISA | IICS Univ de Asuncion |
| Biolab-Merieux | ELISA | Biolab-Mérieux, S.A. |
| c-ELISA (using Bio-Manguinhos kit) | ELISA | Bio-Manguinhos kit |
| rec-ELISA (using Bio-Manguinhos kit) | ELISA | Bio-Manguinhos kit |
| Cypress Chagas Quick Test | RDT | Cypress Diagnostics |
| Operon immunochromatographic test (ICT-Operon; Simple Stick Chagas) | RDT | Operon, S.A. |
| Operon immunochromatographic test (ICT-Operon Simple Chagas WB [whole blood]) | RDT | Operon, S.A. |
| Trypanosoma Detect MRA rapid test (Replaced by Chagas Detect) | RDT | InBios International, Inc. |
| Chagas Instantest | RDT | Silanes |
| Chagas Quick Test | RDT | Cypress Diagnostics |
| Immu-Sure Chagas (T. Cruzi) | RDT | Millennium Biotech |
| Simple Chagas WB | RDT | Operon, S.A. |
| Serodia Chagas | RDT | Fujirebio, Inc. |
| ImmunoComb II Chagas Ab | RDT | Orgenics |
| PATH-Lemos rapid test (Prototype) | RDT | Laboratórios Lemos S.R.L. |
| Abbott PRISM Chagas | CMIA | Abbott |
| CMIA Architect Chagas (chemiluminescent microparticle immunoassays (CMIA) | CMIA | Abbott |
| Teste Chagas-HAI | IHA | Ebram |
| Chagas Hemagen HA | IHA | Hemagen Diagnostics, Inc. |
| Hemacruzi | IHA | Biolab-Mérieux, S.A. |
| IMUNOCRUZI | IIF | Biolab-Mérieux, S.A. |
| TESAcruzi | Western Blot | Biolab-Mérieux, S.A. |
