# Supplementary material for: In vitro diagnostic methods of Chagas disease in the clinical laboratory: a scoping review
Source: Front Microbiol. 2024 Apr 30;15:1393992. doi: 10.3389/fmicb.2024.1393992 (PMC11091413; doi:10.3389/fmicb.2024.1393992)
Supplement: Supplementary file 4 [file Table_4.docx]

**Supplemental Table 4. Summary of In-house Serological Test types**

| **Test** | **Method** |
| --- | --- |
| (Machado et al., 2023). In-house ELISA with rTC (recomb protein antigen) | ELISA |
| (Schaumburg et al., 2023). In-house ELISA-based POCT with Smartphone app | ELISA |
| (Santos et al., 2022). In-house ELISA Chimera IBMP 8.1 | ELISA |
| (Santos et al., 2022). In-house ELISA Chimera IBMP 8.2 | ELISA |
| (Santos et al., 2022). In-house ELISA Chimera IBMP 8.3 | ELISA |
| (Santos et al., 2022). In-house ELISA Chimera IBMP 8.4 | ELISA |
| (Peverengo et al., 2021). In-house ELISA Capture IgM | ELISA |
| (Ferreira-Silva et al., 2021). ELISA-IgG1 and ELISA-IgG3 using Wiener 3.0 | ELISA |
| (Ferreira-Silva et al., 2021). ELISA-rCRP | ELISA |
| (Castro-Sesquen et al., 2021b). Immunoglobulin M (IgM)-Shed Acute Phase Antigen (SAPA) test | ELISA |
| (Mucci et al., 2017). In-house ELISA | ELISA |
| (Reis-Cunha et al., 2014). In-house ELISA (with rTc_11623.20 and rTc_N_10421.310 proteins) | ELISA |
| (Reis-Cunha et al., 2014). In-house ELISA | ELISA |
| (Ferreira et al., 2001). TcF-ELISA | ELISA |
| (Carvalho et al., 1993). In-house (CRA + FRA) *cytoplasmic repetitive antigen (CRA) and flagellar repetitive antigen (FRA) | ELISA |
| (Valdez et al., 2016). In-house ELISA/WB | ELISA/WB |
| (Santos et al., 2018). In-house ELISA Chimera IBMP 8.1 | ELISA |
| (Santos et al., 2018). In-house ELISA Chimera IBMP 8.2 | ELISA |
| (Santos et al., 2018). In-house ELISA Chimera IBMP 8.3 | ELISA |
| (Santos et al., 2018). In-house ELISA Chimera IBMP 8.4 | ELISA |
| (Gamboa-León et al., 2011). In-house ELISA using strain of *T. cruzi* that is prevalent in Mexico | ELISA |
| (Almeida et al., 1997). In-house chemiluminescent ELISA | ELISA |
| (Brashear et al., 1995). In-house Chagas antibody EIA | ELISA |
| (Daltro et al., 2022). In-house Western blot | Western Blot |
| (Ferreira-Silva et al., 2021). TESA-blot | Western Blot |
| (Castro-Sesquen et al., 2021a). IgG-TESA-blot | Western Blot |
| (Kim et al., 2019). Fragmented Ribosomal P protein | Western Blot |
| (Mita-Mendoza et al., 2018). TESAblot | Western Blot |
| (Reiche et al., 1998). In-house Western Blot | Western Blot |
| (Leiby et al., 2000). In-House radioimmunoprecipitation assay (RIPA) | RIPA |
